# Human Bone Marrow Endothelial Progenitor Cell Transplantation into Symptomatic ALS Mice Delays Disease Progression and Increases Motor Neuron Survival by Repairing Blood-Spinal Cord Barrier

**DOI:** 10.1038/s41598-019-41747-4

**Published:** 2019-03-27

**Authors:** Svitlana Garbuzova-Davis, Crupa Kurien, Edward Haller, David J. Eve, Stephanie Navarro, George Steiner, Ajay Mahendrasah, Surafuale Hailu, Mohammed Khatib, Kayla J. Boccio, Cesario V. Borlongan, Harry R. Van Loveren, Stanley H. Appel, Paul R. Sanberg

**Affiliations:** 1 Center of Excellence for Aging & Brain Repair, University of South Florida, Morsani College of Medicine, Tampa, Florida 33612 USA; 2 Department of Neurosurgery and Brain Repair, University of South Florida, Morsani College of Medicine, Tampa, Florida 33612 USA; 3 Department of Molecular Pharmacology and Physiology, University of South Florida, Morsani College of Medicine, Tampa, Florida 33612 USA; 4 Department of Pathology and Cell Biology, University of South Florida, Morsani College of Medicine, Tampa, Florida 33612 USA; 5 Department of Integrative Biology, University of South Florida, Tampa, Florida 33620 USA; 6 Stanley H. Appel Department of Neurology, Houston Methodist Neurological Institute, Houston, Texas 77030 USA; 7 Department of Psychiatry, University of South Florida, Morsani College of Medicine, Tampa, Florida 33612 USA

## Abstract

Convincing evidence demonstrated impairment of the blood-spinal cord barrier (BSCB) in Amyotrophic Lateral Sclerosis (ALS), mainly by endothelial cell (EC) alterations. Replacing damaged ECs by cell transplantation is a potential barrier repair strategy. Recently, we showed that intravenous (iv) administration of human bone marrow CD34^+^ (hBM34^+^) cells into symptomatic ALS mice benefits BSCB restoration and postpones disease progression. However, delayed effect on motor function and some severely damaged capillaries were noted. We hypothesized that hematopoietic cells from a restricted lineage would be more effective. This study aimed to establish the effects of human bone marrow-derived endothelial progenitor cells (hBMEPCs) systemically transplanted into G93A mice at symptomatic disease stage. Results showed that transplanted hBMEPCs significantly improved behavioral disease outcomes, engrafted widely into capillaries of the gray/white matter spinal cord and brain motor cortex/brainstem, substantially restored capillary ultrastructure, significantly decreased EB extravasation into spinal cord parenchyma, meaningfully re-established perivascular astrocyte end-feet, and enhanced spinal cord motor neuron survival. These results provide novel evidence that transplantation of hBMEPCs effectively repairs the BSCB, potentially preventing entry of detrimental peripheral factors, including immune/inflammatory cells, which contribute to motor neuron dysfunction. Transplanting EC progenitor cells may be a promising strategy for barrier repair therapy in this disease.

## Introduction

The blood-brain and blood-spinal cord barriers (BBB and BSCB) are specialized assemblies of microvasculature in the brain and spinal cord maintaining homeostasis in the central nervous system (CNS) by regulating traffic of materials in and out of the systemic compartment and restricting free entry of hazardous blood solutes into the tissues^1–5^. The barrier in the CNS is composed of endothelial cells (ECs) and their tight/adherens junctions, pericytes, and surrounding basement membrane and astrocytic end-feet. Astrocyte processes connect microvessels to the neurons composing the neurovascular unit^6–8^. This unique composition of the BBB/BSCB allows intake of required substances and outtake of metabolic waste products^4,5,9,10^, preserving a CNS environment conducive to proper neuronal cell function. Although the BBB and BSCB share similar structural and functional characteristics, various BSCB physiological differences, i.e. glycogen capillary deposits, greater capillary permeability, and lower expression of tight junction proteins, have been noted^11^. Regardless of these barrier discrepancies, impairment of any barrier component may compromise BBB/BSCB integrity and barrier damage is a potential pathogenic factor in several neurodegenerative diseases^9,12–14^.

During the last decade, convincing evidence of BBB and BSCB impairment has been identified in amyotrophic lateral sclerosis (ALS), a motor neuron disorder. Primarily, alterations of capillary ECs, astrocyte end-feet processes, expression of tight junction proteins, and microvascular permeability were found in the CNS areas of motor neuron degeneration in ALS patients^15–17^ and in animal models of disease^18–23^. Also, Winkler *et al*.^17^ demonstrated significant reduction of pericyte numbers and substantial erythrocyte extravasation in the cervical spinal cords from ALS patients, confirming BSCB disruption. The BBB/BSCB damage, an added element in ALS pathogenesis, should classify ALS as a neurovascular disease^24,25^. Moreover, molecular biomarkers of disease at barrier level are discussed^26,27^. Thus, an impaired barrier, allowing entry of detrimental effectors from the systemic circulation to the CNS, could aggravate degeneration of motor neurons in ALS and may even trigger disease related pathological events^28^.

Although ALS patients exhibit barrier pathologies distinct from the G93A mouse model^29^, dysfunctional capillary endothelial cell lining, downregulation of tight junction proteins, and capillary leakage are shared pathogenic barrier alterations. Cell administration to repair the altered BBB/BSCB may be a promising therapeutic approach for ALS. We recently demonstrated^30^ a dose-response effect from unmodified human bone marrow CD34^+^ (hBM34^+^) cells intravenously administered into symptomatic ALS mice, resulting in delayed disease progression, preserved motor neuron survival, lessened macro- and microgliosis, maintained perivascular astrocyte end-feet, and reduced permeability of spinal cord capillaries. Notably, the transplanted cells were shown to differentiate into ECs and engraft within various capillaries of the spinal cord. Also, in our later study^31^, significantly restored ultrastructural capillary morphology, improved basement membrane integrity, enhanced axonal myelin coherence, and stabilized capillary density in the spinal cords were determined, primarily in ALS mice receiving the high dose of 1 × 10^6^ cells. Additionally, significantly decreased microhemorrhages were noted in spinal cords from ALS mice treated with the hBM34^+^ cells^32^. These results support the benefit of an optimal dose of bone marrow hematopoietic stem cells in repairing the damaged BSCB by delaying disease and preserving motor neuron survival in symptomatic ALS mice. However, the most positive effect of hBM34^+^ cell treatment on motor function in G93A mice was determined 4 weeks after transplantation^30^. Also, a substantial number of severely damaged capillaries was detected via ultrastructural analysis in the cervical and lumbar spinal cords even after a high dose transplant of hBM34^+^ cells^31^. Potentially, administration of restricted-lineage endothelial progenitor cells would provide superior BSCB restoration in ALS.

The goal of this study was determining effects of systemically administered human bone marrow-derived endothelial progenitor cells (hBMEPCs) into symptomatic G93A SOD1 mice. First, the efficacy of cell treatment in regard to measures of animal motor function and motor neuron survival was evaluated. Second, transplanted cell engraftment within microvessels was examined. A specific focus was determining the structural and functional BSCB integrity in hBMEPC-treated mice.

## Methods

### Ethics statement

Our standardized ethics statement follows: “All described procedures were approved by the Institutional Animal Care and Use Committee at USF and conducted in compliance with the *Guide for the Care and Use of Laboratory Animals*. All mice were housed in a temperature-controlled room (23 °C) and maintained on a 12:12 h dark: light cycle (lights on at 06:00 AM). Food and water were available *ad libitum*. Upon progression of neurological symptoms, a highly palatable liquid nutritional supplement was placed on the cage floor, ensuring access by the animal^30–32^”.

### Animals

All study mice were acquired from The Jackson Laboratory, Bar Harbor, ME, USA. Forty-nine 7-week-old transgenic male B6SJL-Tg(SOD1*G93A)1Gur/J mice, a strain which over-expresses human SOD1 by carrying the Gly93 → Ala mutation (G93A SOD1), were randomly assigned to one of two groups receiving human bone marrow endothelial progenitor cells (hBMEPCs) or media: *Group 1* - hBMEPCs (1 × 10^6^ cells/mouse, n = 30) and *Group 2* **-** Media (n = 19). Mouse body weight was measured weekly, beginning at 8 weeks of age, to monitor health. Hindlimb tremor is a common initial symptom of disease in G93A mice, with later reductions in body weight and extension reflex appearing by 12–13 weeks of age^30^, considered as the early symptomatic disease stage. Upon appearance of initial symptoms, ALS mice intravenously (iv, jugular vein) were given hBMEPCs or the same volume of media at 13 weeks of age. *Group* 3 mice, non-transplant controls (n = 24), were animals from the background strain not carrying the mutant SOD1 gene. Mice were again monitored weekly from 14 through 17 weeks of age for symptoms of disease progression.

### Cell preparation and transplant procedure

Cryopreserved human bone marrow-derived endothelial progenitor cells (hBMEPCs) were purchased from CELPROGEN (Torrance, CA, USA). The company reported that cells were obtained from adult donors and that cells were negative for the various viruses and microbial growths screened for via an infectious disease panel. The manufacturer also reported detecting cell markers for CD15 (SSEA-1), CD90, CD105, CD106, CD117, and CD309. Additionally, hBMEPCs were cultured in a 24-well plate (2 × 10^4^ cells/500 μL commercial basal media/well) for 24 hours and fixed by 4% paraformaldehyde in phosphate buffer saline (PBS) solution for immunocytochemical validation of human specific endothelial marker.

Preparation of hBMEPCs for transplantation was performed similarly to our previously described protocol for administration of CD34^+^ cells^30,31^. Cell viability was assessed using the 0.4% trypan blue dye exclusion method before transplantation. Viability of hBMEPCs used for administration was 96.75 ± 1.26% (92.3–100% range). Concentration of cells was adjusted to 5,000 cells/μL (1 × 10^6^ cells/200 μL/injection) prior to transplantation.

The hBMEPCs were delivered via the jugular vein of mice under anesthesia with isofluorane (2–5% at 2 L O_2_/min) as we previously described^33,34^ with minimal modifications^30,31^. Group 2, Media mice, received 200 μL of Dulbecco’s Phosphate Buffered Saline 1 × (DPBS), equivalent to the cell-transplanted-mice volume. Animals in Groups 1 and 2 received cyclosporine A (CsA, 10 mg/kg ip) daily for the entire post-transplant period.

### Characteristics of disease progression

We have previously detailed methods to evaluate disease progression in mice^30,33–35^. To provide unbiased evaluations, behavioral testing was conducted by technicians blinded to animal status. Mouse body weight was measured each week. Tests of extension reflex, rotarod, and grip strength tests began at age 8 weeks, repeating weekly through age 17 weeks.

### Perfusion and tissue preparation

All hBMEPC-treated, media, and control animals were sacrificed at age 17 weeks (4 weeks post-cell or media administration) for immunohistochemical, ultrastructural (electron microscopy), and histological analyses of cervical and lumbar spinal cords. Animal sacrifices at 17 weeks of age replicated our earlier reports^30,31^ and this age is close to the disease’s end stage. The G93A (Group 1: n = 10 and Group 2: n = 9) and control mice (Group 3: n = 9) received 2% Evans Blue dye (EB, Sigma-Aldrich, St. Louis, MO, USA) in a saline solution (4 mL/kg body weight) by tail vein at 30 min prior to perfusion^30^. Before the perfusion, blood samples (500–700 μL) were obtained by cardiac puncture from randomly selected hBMEPC-treated, media, and control animals (n = 5–7/group). Collected blood was placed into serum separation tubes (Corvac^TM^) for 10 minutes at RT and then centrifuged at 1200 rpm for 15 minutes to obtain sera.

Mice were sacrificed under Euthasol® using previously described perfusion techniques^30,31^. Mice assayed for EB extravasation received only PBS and upon perfusion, the entire spinal cords were rapidly removed from hBMEPC-treated (n = 7), media (n = 5) and control mice (n = 6) to examine EB extravasation^30,31^. In the remainder of the animals (n = 3–4/group) injected with EB, segments of cervical and lumbar spinal cord were removed, post-fixed, cryopreserved, and cut in a cryostat as described^30,31^. Collected coronal spinal cord tissues were stored for immunohistochemistry analysis of microvessel EB at −20 °C. Also, mice (Group 1, n = 15; Group 2, n = 7; Group 3, n = 12) were perfused and the spinal cords were excised, post-fixed, cryopreserved, and cut as described above for later examinations by immunohistochemistry and histology. Similarly, brains were removed, post-fixed, cryoprotected, and sliced longitudinally at 30 μm for immunohistochemistry. Also, during perfusion, blood was collected from randomly selected hBMEPC-treated (n = 8) and media ALS animals (n = 3). The obtained blood smears were then fixed in methanol for 10 min for later immunocytochemistry tests.

Animals for electron microscopy (EM) examinations were randomly selected from hBMEPC-treated, media, and control groups (n = 3–4/group). Cervical and lumbar spinal cords were processed for testing as described^31^. Morphology of cervical and lumbar spinal cord capillaries in areas of anterior motor neurons were examined by EM as detailed^31^.

### BSCB integrity analysis

Microvessel ultrastructure in the cervical and lumbar spinal cords of cell-treated, media-treated, and control mice was examined by an investigator blinded to the animal groups, using coded sections as described^31^. Microvessel morphologies of hBMEPC-treated, media, and control spinal cords were examined from EM images. Numbers of examined microvessels were: *the cervical spinal cord* – controls (n = 54), media (n = 78), and hBMEPC-treated (n = 40); *the lumbar spinal cord* – controls (n = 68), media (n = 90), and hBMEPC-treated (n = 40). Capillaries were separated into three categories on the basis of *normal*, *moderately impaired*, *or severely compromised morphology*. Characteristics of capillary morphology were mainly based on endothelium, perivascular cells, and neuropil status as described in detail^31^.

### BSCB permeability

BSCB permeability was determined using Evans blue (EB) dye, 961 Da. This examination of EB extravasation was detailed in our prior articles^19,30,31^.

### Immunohistochemistry and immunocytochemistry

Characteristics of EB leaks from microvessels were determined in a series of tissue sections from EB injected mouse spinal cords (n = 3–4/group) as previously described^31^. In a separate set of the spinal cord tissue sections, immunohistochemical staining for CD31 was performed to visualize capillary permeability for EB. Briefly, the rat monoclonal anti-CD31 antibody (1:50, RM0032-ID12, catalog number ab56299, Abcam, USA) was applied on tissues after pre-incubation in a blocking solution (10% normal goat serum/3% Triton 100×/PBS) for 60 min at room temperature (RT). The next day, tissue sections were rinsed in PBS and incubated with goat anti-rat secondary antibody conjugated to rhodamine (1:700, catalog number ab150157, Abcam, USA) for 2 hrs at RT. The slides were then rinsed with PBS and coverslipped with Vectashield containing DAPI (Vector Laboratories, USA). The tissue sections were examined under epifluorescence using an Olympus BX60 microscope and images were taken for further analysis.

In a different collection of spinal cord (cervical and lumber) and brain tissue sections from randomly selected hBMEPC-treated (n = 7) and media mice (n = 3), human anti-Von Willebrand Factor (vWF), an endothelial cell marker, was used to determine engraftment of transplanted cells via immunohistochemical staining in microvessels as previously detailed^30^. Obtained images were stored for later examination of vWF fluorescent immunoexpression. Additionally, blood smears from ALS mice (n = 8) and media-treated mice (n = 3) were immunostained with human anti-vWF as described^30^. Analysis of immunopositive cells for vWF in blood smears from treated ALS mice were manually enumerated from the complete slide at 40X and presented as percentages of total nucleated cells. To validate human anti-vWF *in vivo*, hBMEPCs were cultured for 24 hours prior to performing identical immunocytochemical staining, including marker concentration as described^30^. Fluorescent *in vitro* images were then obtained for later examination.

In serial spinal cord sections from randomly selected hBMEPC-treated, media, and control animals (n = 5/group), immunohistochemical staining for astrocytes via GFAP was performed as we have detailed^30^.

### vWF and perivascular astrocytic analyses in the spinal cord

Immunoexpressions of vWF and GFAP fluorescences in the cervical and lumbar spinal cord ventral horns from mice at 17 weeks of age were analyzed via NIH ImageJ software by a technician blinded to the experiments as we previously detailed^30^. Cells immunopositive for vWF were detected in hBMEPC-treated mice using immunohistochemical images (n = 7/group, n = 6–11images/spinal cord segment) from right and left ventral gray matter of the cervical and lumbar spinal cords at 40×. The complete image was examined for intensity (%/mm^2^) of fluorescence. Results are indicated as average immunoexpression in vWF cells from both spinal cord sides. Immunohistochemical images for vWF immunoexpressions in brain tissues from randomly selected hBMEPC-treated (n = 3) and media (n = 3) mice were analyzed in motor cortex (M2/M1) and brainstem (pons, medulla) areas according to a mouse brain atlas^36^.

Immunoexpression of GFAP in the perivascular space was examined in cervical and lumbar ventral horns from both sides of hBMEPC-treated, media-injected, and control animals. Strength of GFAP fluorescence in astrocyte end-feet (perivascular astrocytes) was examined adjacent to abluminal side of microvessels roughly 27 µm in diameter (n = 5 mice/group, n = 12–18 microvessels/segment of the spinal cord). Perivascular GFAP immunoexpression densities were presented as percentages per mm^2^ independently for both cervical and lumbar spinal cord sites. Additionally, reactive and non-reactive astrocytic morphologies were identified. Cell bodies of reactive astrocytes are larger, with thicker, and more clearly visible processes, as compared to the thin processes of non-reactive astrocytes.

### Histological staining and stereological counting of motor neurons in the spinal cord

Different cervical and lumbar spinal cord sections from randomly selected animals from each group (n = 5/group) were stained with 0.1% cresyl violet to analyze motor neuron condition for Nissl substance. Counts of motor neurons from cervical and lumbar spinal cord ventral horns were obtained through stereological techniques as we previously detailed^30^. Motor neurons (~23 μm diameter) were enumerated in distinct cervical and lumbar spinal cord segments (n = 10–14 sections/level/spinal cord segment/group separated by roughly 120 μm) according to a mouse spinal cord atlas^37^.

### Statistical analysis

Data presentation and statistical analysis were described previously as: “Data are presented as means ± S.E.M. One-way ANOVA with post-hoc Tukey HSD (Honesty Significant Difference) multiple comparison test using online statistical software (astatsa.com, 2016 Navendu Vasavada) was performed for statistical analysis. Significance was defined as p < 0.05^31^”.

## Results

The hBMEPCs were systemically transplanted into 13-week-old symptomatic G93A SOD1 mice. The mice were sacrificed at 17 weeks of age, 4 weeks post-transplant. Forty nine G93A SOD1 mice entered into the study and three of these animals (Group 1 – two, Group 2 – one) were removed due to non-disease-related deaths, resulting from anesthetic complications during transplant procedures. Also, three mice (Group 1 – two, Group 2 – one) were found dead at 16 or 17 weeks of age; data from behavioral testing of these mice remained in our analyses.

### Effect of hBMEPC transplantation on disease outcomes

**Figure 1:**
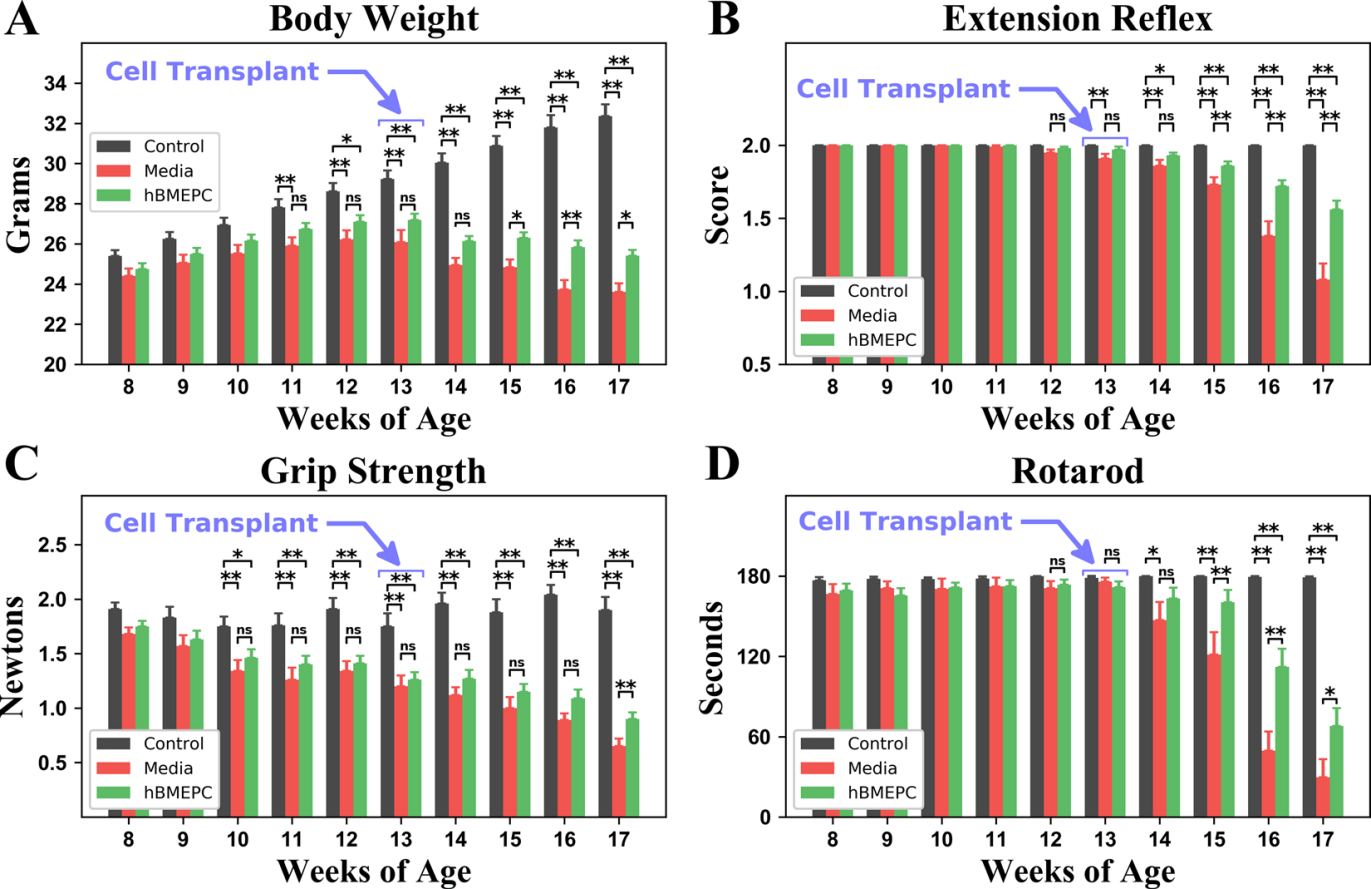
Characteristics of disease outcomes in G93A mice receiving hBMEPCs at symptomatic stage. Transplanted ALS mice with cell dose of 1 × 10^6^ at 4 weeks post-treatment (**A**) significantly maintained body weight, (**B**) better extended hindlimbs, (**C**) delayed loss in muscle strength, and (**D**) stayed longer on rotarod vs. media-injected mice. Notable beneficial effects on motor function were determined in G93A mice 2–3 weeks after cell transplantation. ^*^p < 0.05, ^**^p < 0.01.

Body weight, an indicator of health and progressive muscle atrophy, was measured each week. As is typical, mouse body weights began to gradually decline at approximately 13–14 weeks of age in media G93A mice. By 17 weeks of age, these mice retained about 90% of their maximum body weights. The hBMEPC-treated mice better maintained body weight, yet one week after transplant no significant differences were noted between media and cell-treated animals. At two weeks after transplant, cell-treated animals had achieved significantly (p = 0.027) higher body weights versus media mice (Fig. 1A). Three and four weeks post-transplant, cell-treated animals maintained their significantly higher body weights (16 weeks of age: p = 0.009; 17 weeks of age: p = 0.021). Notably, body weights of 17-weeks-of-age cell-treated mice were approximately 2 grams higher than media mice.

Mice receiving hBMEPCs also better performed tests of functional ability. Starting at 12–13 weeks of age, worsening extension reflex scores were noted in media G93A mice (1.95 ± 0.02 and 1.91 ± 0.03 scores, respectively), with scores continuing to drop until 17 weeks of age (1.08 ± 0.11 score). However, extension scores of cell-treated animals declined at a slower rate than scores of media-injected mice. Although at one week post-cell-transplant ALS mice tended towards delayed deterioration of hindlimb extension scores versus media animals, significantly higher extension reflex scores vs. media mice were demonstrated at 15 (p = 0.007), 16 (p = 0.001), and 17 (p = 0.001) weeks of age (Fig. 1B).

In the grip strength test, G93A mice receiving media indicated a decline in muscle strength by approximately 13 weeks of age (1.20 ± 0.10 N), with losses progressing over the course of disease until 17 weeks of age (0.65 ± 0.07 N) (Fig. 1C). In contrast, these grip declines were delayed in animals receiving hBMEPCs versus media beginning at 14 weeks of age and reaching significance (p = 0.003) at 17 weeks of age (Fig. 1C).

Rotarod performance declines were also noted in media mice from week 12. Animals receiving hBMEPCs showed longer latencies than media mice. Significantly higher rotarod latencies were noted in ALS mice receiving cell transplantation at 15 (p = 0.004), 16 (p = 0.001), and 17 (p = 0.036) weeks of age corresponding to 2, 3, and 4 weeks post-transplant, respectively (Fig. 1D). Importantly, at 17 weeks of age, hBMEPC-treated mice showed notably higher rotarod latency (68.06 ± 13.21 sec) compared to media animals (29.60 ± 13.57 sec).

Thus, intravenous transplantation of hBMEPCs into early symptomatic G93A mice led to obvious improvements in post-treatment behavioral outcomes compared to media-injected mice through maintained body weight and delayed reductions in hindlimb extension, grip strength, and rotarod performance. Notably, these benefits to motor function were determined 2–3 weeks after cell transplantation into G93A mice.

### Immunocytochemical analysis of hBMEPCs *in vitro*

Immunocytochemical staining for human anti-Von Willebrand Factor (vWF) of hBMEPCs cultured for 24 hours showed antigen immunoexpression in all cells (see Supplementary Fig. 1S). However, different degrees and localization of vWF were observed according to cell morphology. Rounded cells had high levels of vWF immunoexpression. Elongated cells displayed different cellular vWF locations at perinuclear space, cell tip, or near inner cell membrane, potentially reflecting dissimilar profiles of vWF antigen expression during cell development *in vitro*. Importantly, this study provides evidence that human anti-vWF is an appropriate marker to identify transplanted hBMEPCs *in vivo*.

### Immunohistochemical analyses of transplanted hBMEPCs *in vivo*

**Figure 2:**
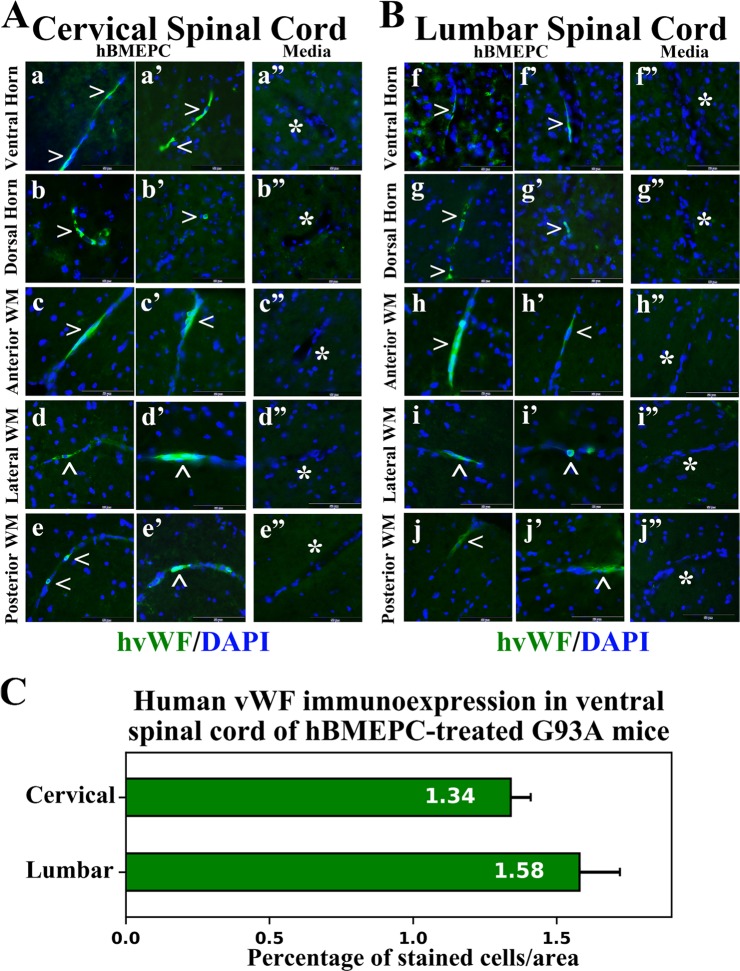
Immunohistochemical staining for human vWF in the cervical and lumbar spinal cords of cell-treated G93A mice. (**A**) In the cervical spinal cord, immunopositive vWF expression (green, arrowheads) was indicated in cells within capillaries of the ventral (a,a’) and dorsal (b,b’) spinal cord horns of cell-treated mice. These adherent transplanted cells expressing vWF in lumen of microvessels formed a distinguishable lining in capillary walls. Positive cellular immunoexpression of vWF (green, arrowheads) was also indicated in anterior (c,c’), lateral (d,d’), and posterior (e,e’) white matter microvessels of the cervical spinal cords. (**B**) Similarly to the cervical spinal cord, transplanted cells expressing vWF (green, arrowheads) indicated their engraftment into vascular lumen in the ventral (f,f’) and dorsal (g,g’) horns, anterior (h,h’), lateral (i,i’), and posterior (j,j’) white matter of the lumbar spinal cords. However, some cells of rounded or oval shaped morphology were noted in lumbar lateral white matter (i’) or cervical dorsal horn (b’) capillaries. No vWF immunoexpression was found in capillaries or parenchyma of the cervical (a”, b”, c”, d”, e”) and lumbar (f”, g”, h”, i”, j”) spinal cords from media-injected mice. Images are merged with DAPI. Scale bar in a–j’ is 50 µm. (**C**) Quantitative analysis of fluorescent vWF expression in ventral gray matter of the spinal cords showed moderately increased intensities of fluorescent expression in the lumbar vs. cervical spinal cord segments.

Four weeks after intravenous cell transplants, cervical and lumbar spinal cord tissues from hBMEPC-treated mice were immunohistochemically stained with human anti-vWF, a marker for endothelial cells. Spinal cord tissues from media-treated mice were also stained for vWF and used as a control. The vWF immunoexpression was noted in cells within the ventral and dorsal spinal cord horns of hBMEPC-treated G93A mice in both cervical (Fig. 2Aa,Aa’) and lumbar (Fig. 2Bf,Bf’) segments. Transplanted hBMEPC cells adhered to microvessel lumen, forming a line on capillary walls of the cell-treated G93A animals and indicating superior hBMEPC engraftment. Immunopositive cellular expression of vWF was also indicated in anterior (Fig. 2Ac,Ac’), lateral (Fig. 2Ad,Ad’), and posterior (Fig. 2Ae,Ae’) white matter microvessels within cervical spinal cords. Similarly, transplanted cells immunopositive for vWF were found in examined anterior (Fig. 2Bh,Bh’), lateral (Fig. 2Bi,Bi’), and posterior (Fig. 2Bj,Bj’) capillaries of lumbar spinal cord white matter. Although most cells stained for vWF were found in the lining of the lumen of microvessels in analyzed gray and white matter spinal cord areas, some cells were rounded or oval shaped in the cervical dorsal horn (Fig. 2Ab’) or lumbar lateral white matter (Fig. 2Bi’) capillaries. Notably, no cells expressing vWF were noted in spinal cord parenchyma of hBMEPC-treated animals or in microvessels/parenchyma of the cervical (Fig. 2Aa”–Ae”) and lumbar (Fig. 2Bf”–Bj”) spinal cords of ALS mice receiving media.

Analysis was performed on randomly selected right and left ventral gray matter spinal cord areas to quantify vWF fluorescence, showing moderately higher fluorescent expression in lumbar than cervical segments of the spinal cord (Fig. 2C).

**Figure 3:**
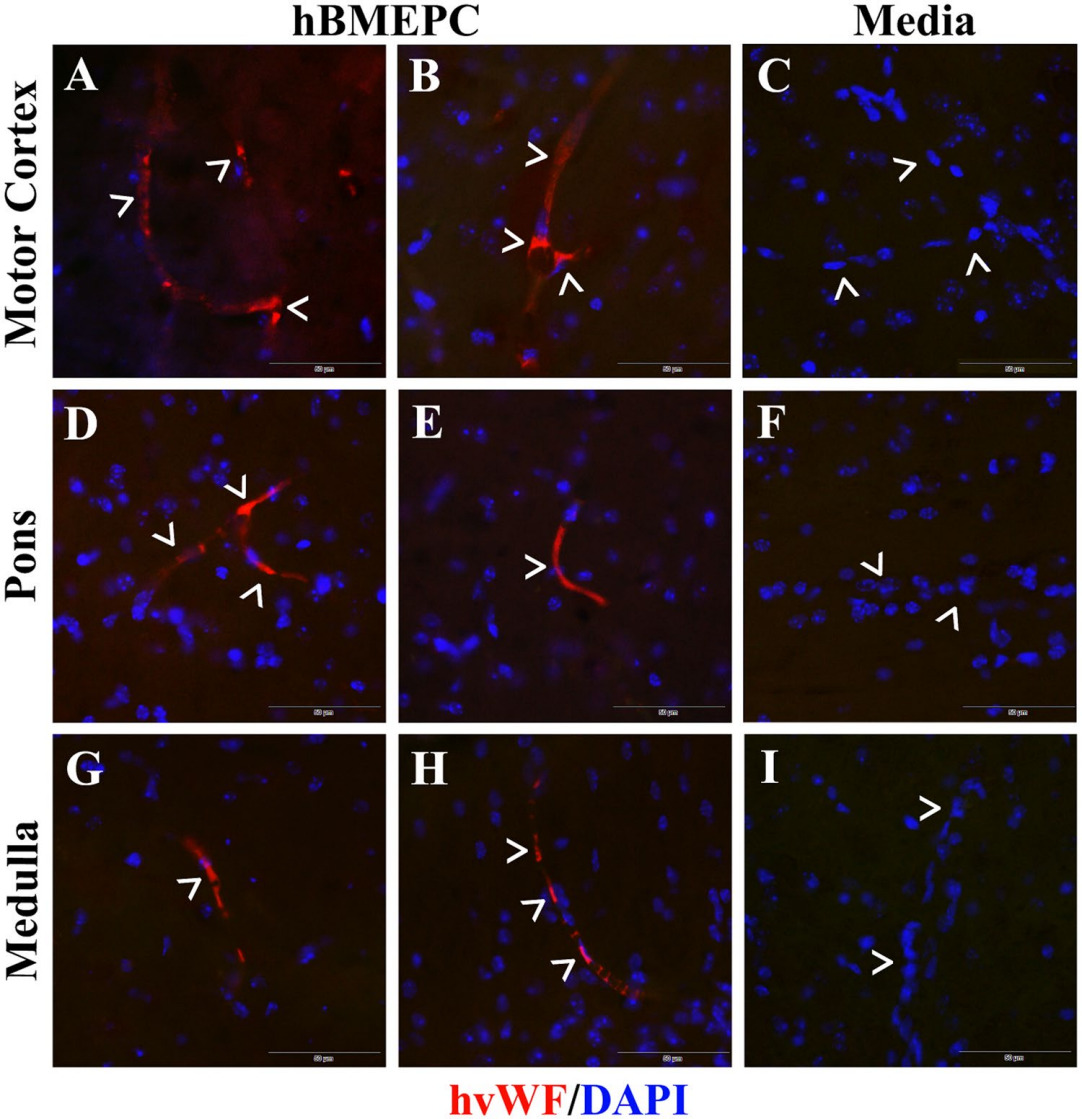
Immunohistochemical staining for human vWF in the brains of cell-treated G93A mice. Immunohistochemical staining of the brain tissues from cell-treated mice with human vWF showed immunopositive cells expressing vWF antigen (red, arrowheads) in numerous capillaries of (**A,B**) motor cortex (M2/M1), (**D,E**) pons, and (**G,H**) medulla. These cells are located within the capillary lumen indicating superior cellular engraftment. No cells expressing vWF were determined in analyzed brain areas (**C,F, I**) from media-injected mice. Images are merged with DAPI. Scale bar in A–I is 50 µm.

Also, transplanted cell immunoexpressions for vWF were analyzed in motor cortex (M2/M1) and brainstem (pons and medulla) in the brains from cell-treated ALS mice. Positive cellular immunoexpression of vWF was determined in motor cortex (Fig. 3A,B), pons (Fig. 3D,E), and medulla (Fig. 3G,H). Cells immunopositive for vWF were visualized in the lumen of numerous brain microvessels. Important, negative vWF immunoexpression was indicated in capillaries or parenchyma of the brains (Fig. 3C,F,I) from media-treated mice, likewise in the spinal cords from the same mice.

**Figure 4:**
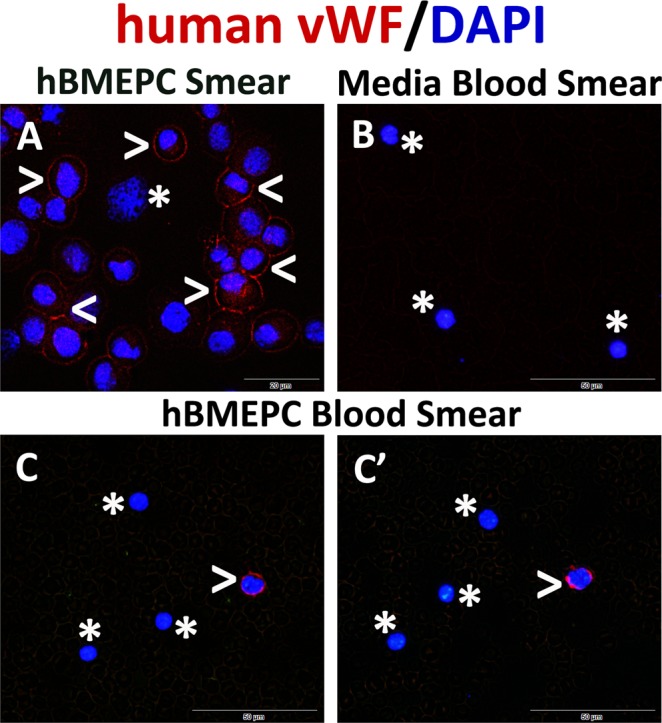
Immunocytochemical analysis of transplanted hBMEPCs in blood smears. For identification of transplanted hBMEPCs within blood circulation of treated mice, immunofluorescent staining with the human anti-vWF was performed in blood smears. (**A**) hBMEPCs were positive for vWF immunoexpression (red, arrowheads). A few cells did not express vWF (asterisk) likely due to damaging during smear preparation. (**B**) There was no detection of human cells by vWF marker in blood smears from media-treated mice (asterisks). (**C**, C’) In blood smears from cell-treated ALS mice, some cells were identified with human vWF (red, arrowheads). Cells negative for vWF are noted by asterisks. The nuclei in all images are shown with DAPI. Scale bar in A is 20 µm and B-C’ is 50 µm.

Additionally, to identify hBMEPCs in the blood circulatory system, blood smears were stained with human anti-vWF. The hBMEPC smears received identical staining and served as controls. hBMEPCs in smears were immunopositive for vWF (Fig. 4A). Human cells were not detected in blood smears of animals receiving media (Fig. 4B). In blood smears from cell-treated ALS mice, some cells showed immunopositivity for vWF (Fig. 4C,C’). About 10% (9.12 ± 1.52%) of cells were positive for vWF in blood smears from hBMEPC-treated ALS mice.

Therefore, *in vivo* immunohistochemistry revealed engraftment of transplanted cells into capillaries in the gray/white matter spinal cords and brain motor cortex/brainstem by 4 weeks post-transplant, suggesting widespread vascular integration of administered human cells within mouse CNS tissue. Additionally, the presence of transplanted hBMEPCs in blood circulation as confirmed by vWF immunostaining that may support the concept of ongoing processes towards re-established integrity of the BSCB.

### Ultrastructure of spinal cord capillaries

An electron microscope (EM) technique was used to evaluate the ultrastructure of cervical and lumbar spinal cord microvessels of 17-weeks-of-age cell-treated, media, and control animals. In G93A mice, EM images were analyzed 4 weeks post cell transplant or media administration to determine BSCB ultrastructural condition.

#### Cervical spinal cord

**Figure 5:**
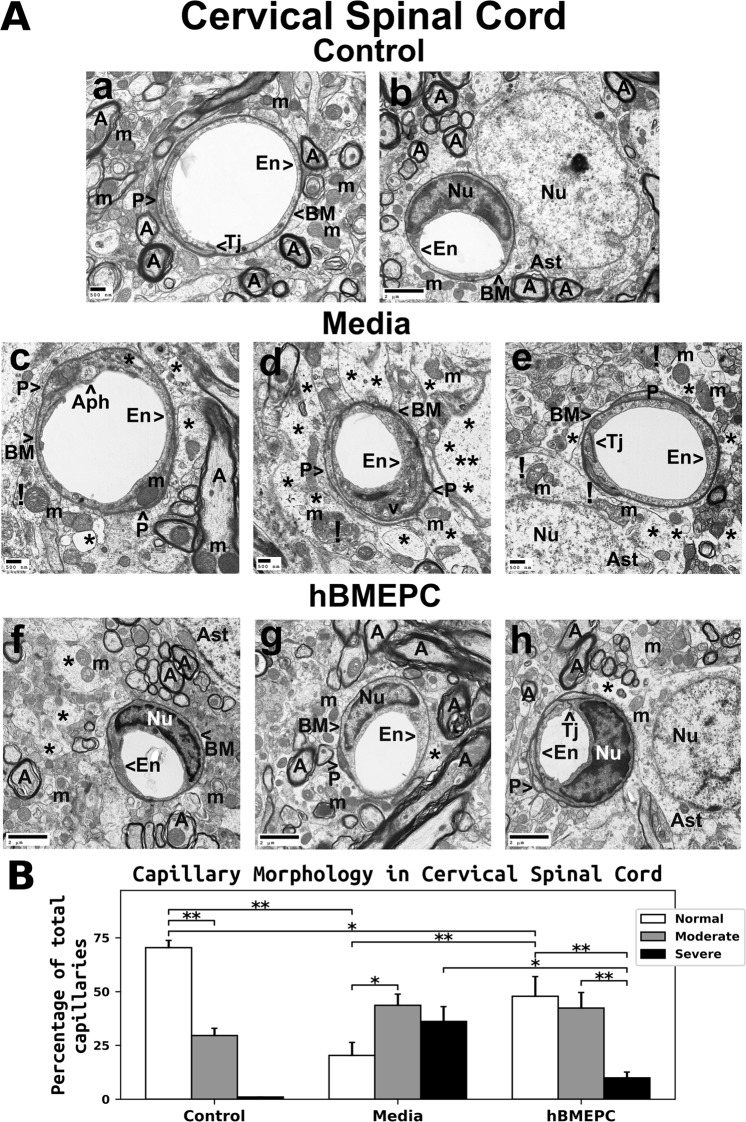
Characteristics of capillary ultrastructure in the cervical spinal cord of G93A mice. (**A**) *Electron microscopy examination of microvasculature in the cervical ventral horn*. Control mouse showed typical ultrastructure of endothelium, adjacent pericytes, tight junctions, neuropil, and axons (a,b). Capillary consisted of an endothelial cell and a single layer of basement membrane surrounding by astrocyte foot processes. Mitochondria and myelinated axons were well preserved. In media-treated mice at 17 weeks of age, swollen or vacuolated endothelial cells (c,d), degenerated astrocyte foot-processes (e), and extensive perivascular protein-filled edema (d) were determined in numerous capillaries. Large autophagosome (a) was found in cytoplasm of endothelial cell. Some capillaries showed pericytes with small cell processes (d). (c,d,e) Swollen and/or degenerated free floating mitochondria indicated by (**!**) and degenerated myelinated axons were noted. (f,g,h) Numerous capillaries demonstrated typical endothelium morphology, pericytes, and perivascular astrocytes in mice after cell treatment. Multiple pericyte cell extensions (h) were visible under capillary basement membrane. Some areas of perivascular edema between capillary and astrocyte end-feet (f,h) and reduced axonal myelin (g) were determined. Capillary tight junction was well defined (h). En – endothelial cell, Tj – tight junction, BM – basement membrane, P - pericyte, Aph – autophagosome, Ast – astrocyte, A – axon, m – mitochondrion, v – vacuole, Nu – nuclei, * - perivascular edema, ! - swollen and/or degenerated mitochondria. Scale bar in a, c, d, e is 500 nm; b, f, g, h is 2 µm. **(B)** *Quantitative analysis of capillary morphology in the cervical ventral horn*. Control mice showed a high percentage of capillaries with normal morphology and low numbers of moderately impaired capillaries. Media mice demonstrated a significant decrease of morphologically normal capillaries, an increase of capillaries with moderate impairment, and a high percentage of severely damaged microvessels. Cell-treated mice displayed a significant increase of capillaries with normal morphology and a decreased percentage of severely compromised capillaries vs. media-injected animals. However, no significant differences were found between cell-treated and media-treated mice in the percentage of capillaries with moderate morphological impairment. ^*^p < 0.05, ^**^p < 0.01.

Results of EM imaging analysis of cervical spinal cords showed normal morphology in microvessels and perivascular astrocytes (Fig. 5Aa,Ab) of control animals. Analyzed capillaries contained a single layer of endothelial cells (ECs), adjoining tight junctions, and adjacent pericytes. Astrocyte foot processes surrounded the capillary basement membrane. Axons in the neuropil were myelinated and exhibited normal morphology. A typical patterning of cristae was present in the mitochondria.

Substantial aberrations of the ventral spinal horn ultrastructure were seen in ALS mice receiving media (Fig. 5Ac,Ad,Ae). Many capillaries contained swollen ECs with large autophagosomes (Fig. 5Ac) or cytoplasmic vacuolization (Fig. 5Ad). Although pericytes appeared with normal cytoplasm and cell extension surrounded by capillary basement membrane (Fig. 5Ac,Ae), some capillaries displayed pericytes with very small cell processes (Fig. 5Ad). A few swollen/degenerated mitochondria were observed floating freely among remains of degenerated perivascular astrocyte processes or in cell cytoplasm (Fig. 5Ae). Proximal to capillaries, areas of extracellular edema were common (Fig. 5Ad). Axonal myelin had appearance of degeneration. In ALS mice receiving hBMEPCs, many capillaries showed normal morphologies for ECs, pericytes, and perivascular astrocytes (Fig. 5Af,Ag,Ah). Pericytes contained a normal appearing mitochondrion (Fig. 5Ag) and multiple pericyte cell extensions were visible under the basement membrane (Fig. 5Ah). However, a few regions with perivascular edema were noted (Fig. 5Af,Ah). Axons showing evidence of losses to their myelin sheaths were occasionally noted (Fig. 5Ag). Yet tight junctions of capillaries were clearly evident and of normal appearance (Fig. 5Ah).

Capillary morphologies analyzed in the cervical spinal cords of 17-weeks-of-age control mice showed more microvessels of normal morphology (70.40 ± 3.30%) than with modest defects (29.60 ± 3.29%) (Fig. 5B). No capillaries in control animals were observed with severe impairment. ALS mice of the same age that had received media demonstrated significantly (p = 0.001) reduced numbers of capillaries (20.35 ± 6.06%) with normal morphology versus controls and significantly (p = 0.020) increased amount of modestly damaged capillaries (43.55 ± 5.28%) than capillaries with normal appearance. A large portion of microvessels with severe impairment (36.10 ± 6.94%) was noted in mice receiving media (Fig. 5B). The portion of microvessels with normal morphologies was significantly (p = 0.009) elevated in hBMEPC-treated G93A animals to 47.80 ± 9.17% vs. media mice. The fraction of capillaries with severe damage showed a significant (p = 0.013) reduction after cell treatment (9.87 ± 2.72%) compared to media mice (Fig. 5B). However, no significant differences were identified in the percentage of capillaries with moderate morphological impairment between cell-treated (42.33 ± 7.19%) and media-treated animals (43.55 ± 5.28%) (Fig. 5B).

#### Lumbar spinal cord

**Figure 6:**
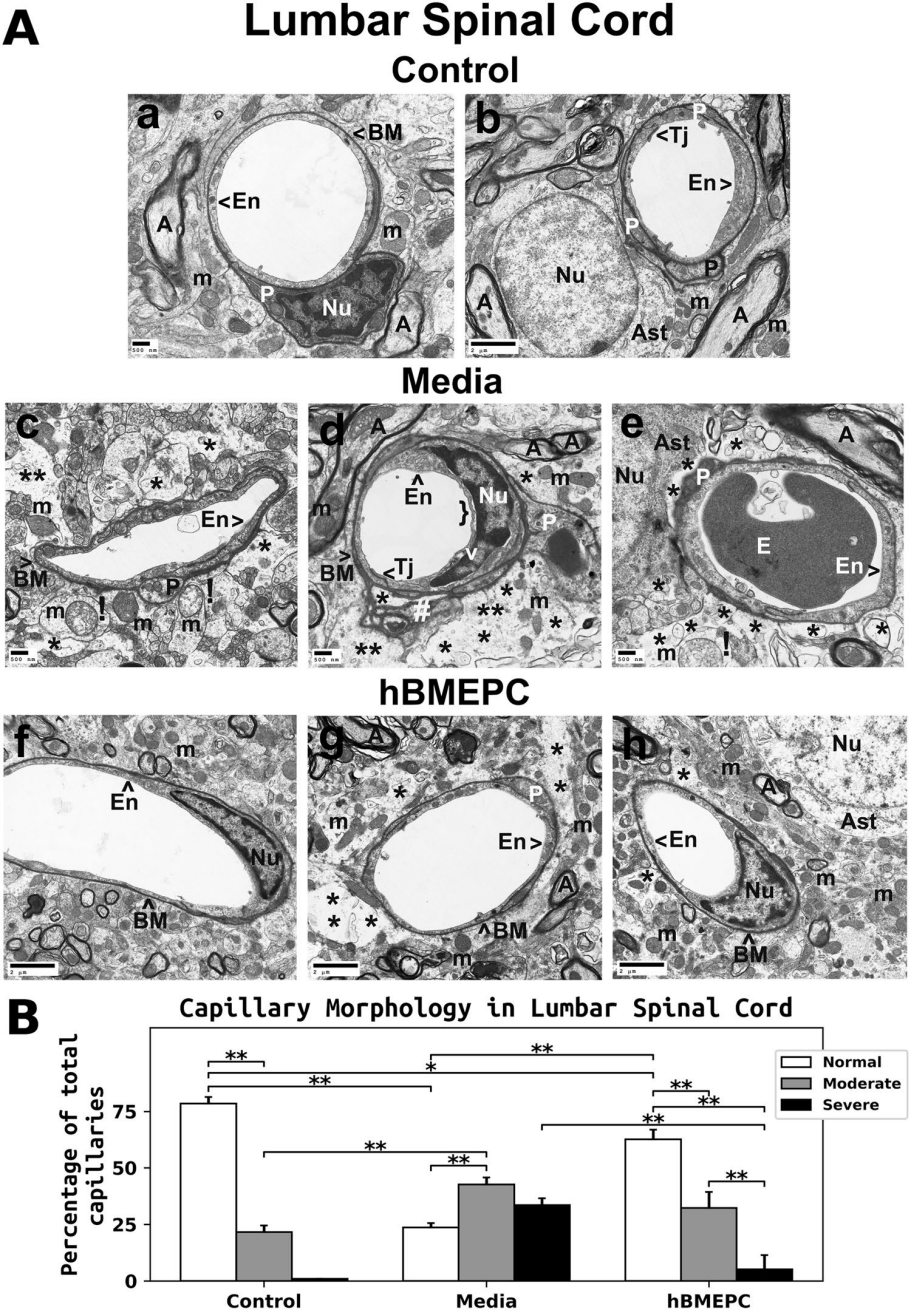
Characteristics of capillary ultrastructure in the lumbar spinal cord of G93A mice. (**A**) *Electron microscopy examination of microvasculature in the lumbar ventral horn*. Control mouse demonstrated intact ultrastructural morphology of lumbar ventral horn capillaries (a,b), similar to findings in the cervical spinal cord. Capillary consisted of typical endothelium composed of a single layer of endothelial cells, pericytes, tight junction, surrounding basement membrane, and adjacent astrocyte end-feet. Several pericyte extensions were noted. Mitochondria and myelinated axons were well preserved in neuropil. (c,d) Media-treated mice demonstrated degenerated endothelial cells and astrocyte foot-processes. Pericytes with a small cell extension were seen (c). (d) A large dark spot in the center of pericyte cytoplasm and numerous vesicles were determined in a tangential section. (e) Pericyte with a condensed cytoplasm was detected. Substantial perivascular protein-filled edema was determined in numerous capillaries. (c,e) Swollen or degenerated mitochondria indicated by (**!**) were also observed in perivascular space. Myelin disruptions or reduced myelin sheathing in surrounding axons were noted. In capillary (d), detachment of luminal endothelial membrane and rupture of basement membrane on extracellular edema side were distinguished. (f,h) A substantial improvement of capillary morphology was detected in cell-treated ALS mice. Normal ultrastructural morphology of ECs and pericytes, intact mitochondria, adjacent astrocyte end-feet, and preserved axonal myelin were detected. (g) Yet, a small pericyte cytoplasmic extension was evident. Although small areas of perivascular edema were seen (h), some capillaries displayed a large area of perivascular edema (g). En – endothelial cell, Tj – tight junction, BM – basement membrane, P - pericyte, Ast – astrocyte, A – axon, m – mitochondrion, v – vacuole, E - erythrocyte, Nu – nuclei, * - perivascular edema, ! - swollen and/or degenerated mitochondria, } - detachment of luminal endothelial membrane, # - rupture of basement membrane. Scale bar in a, c, d, e is 500 nm; b, f, g, h is 2 µm. **(B)** *Quantitative analysis of capillary morphology in the lumbar ventral horn*. Capillary profiles in the lumbar spinal cords were similar to those observed in the cervical spinal cords. Control mice showed a high percentage of capillaries with normal morphology and a low percentage of moderately impaired capillaries. Media-treated mice demonstrated a significant reduction of morphologically normal capillaries, an increase of capillaries with moderate impairment, and a high percentage of severely damaged microvessels vs. controls. A significant increase of normal capillaries was demonstrated in cell-treated mice compare to media-injected mice. The percentage of severely compromised capillaries was significantly reduced in cell-treated mice. A non-significant decrease in the percentage of capillaries with moderate morphological impairment was noted in mice receiving cell transplant vs. media animals. ^**^p < 0.01.

Similar to cervical spinal cord findings, microvessels from the lumbar spinal cord ventral horn of control animals had normal morphology, comprising an EC layer, basement membrane, pericytes, tight junction, and adjoining astrocyte end-feet (Fig. 6Aa,Ab). Pericytes contained typical mitochondria and rough endoplasmic reticulum (Fig. 6Aa) and several cell extensions were noted beneath the capillary basement membrane (Fig. 6Ab). Axonal myelin and neuropil mitochondria seemed normal. ALS animals that had received media displayed swollen or vacuolated ECs in lumen of capillaries, degenerated astrocyte end-feet, and substantial extracellular edema surrounding capillaries (Fig. 6Ac,Ad,Ae). A small pericyte extension with normal appearing mitochondria was shown in capillary (Fig. 6Ac). Figure 6Ad demonstrated a tangential section through a pericyte cell extension and a large dark spot in the center of pericyte cytoplasm was determined. In this image, organelles in the pericyte appeared normal in size and numerous transport vesicles were seen in cell cytoplasm. Similarly, the tangential capillary section through the end of pericyte displayed a condensed cell cytoplasm (Fig. 6Ae). Mitochondrial degeneration was also noted in regions of degenerate perivascular astrocyte end-feet (Fig. 6Ac,Ae). In capillary, detachment of luminal endothelial membrane and rupture of basement membrane on extracellular edema side were noted (Fig. 6Ad). Also, indications of disrupted myelin or reduced myelin sheathing in surrounding axons were found. Contrastingly, substantially improved microvessel morphology was identified in ALS cell-treated mice (Fig. 6Af,Ah). ECs and pericytes showed normal ultrastructure, intact mitochondria, adjoining astrocyte end-feet, and preservation of axonal myelin. However, a small pericyte cytoplasmic extension surrounded by capillary basement membrane was evident (Fig. 6Ag). Although minimal regions of edema were noted in the perivascular space (Fig. 6Ah), some capillaries displayed a large area of perivascular edema (Fig. 6Ag).

Lumbar spinal cord microvessel morphologies shared similarities with cervical spinal cord segment. Control mice exhibited normal capillary morphology (78.43 ± 2.92%) and some microvessels had moderate impairment (21.58 ± 2.90%) (Fig. 6B). No control animals showed severe microvessel damage. Compared to controls, ALS mice receiving media demonstrated a significantly (p = 0.001) lower percentage of microvessels with normal morphology (23.70 ± 1.91%) and a higher percentage of capillaries with moderate damage (42.70 ± 3.05%). A large portion of microvessels from media mice (33.60 ± 2.94%) were determined to have severe impairment (Fig. 6B). A significantly (p = 0.001) higher fraction of normal-morphology capillaries was noted in hBMEPC-treated (62.60 ± 4.33%) versus ALS media mice. The fraction of capillaries with severe impairment showed a significant (p = 0.001) reduction to 5.13 ± 6.29%, although a large variation in capillary counts was noted due to two mice showing no evidence of capillaries with severe morphology (Fig. 6B). However, a non-significant reduction in the portion of capillaries with moderate morphological impairment in cell-treated (32.27 ± 7.06%) vs. media-treated (42.70 ± 3.05%) animals was determined (Fig. 6B).

Together, the results demonstrated substantial damage of BSCB in the cervical and lumbar spinal cord ventral horns of 17-weeks-old ALS media mice. Structural barrier restoration in the spinal cord was noted in animals transplanted with hBMEPCs and was confirmed by microvessel morphologies, specifically, by significantly increased fractions of typical-morphology capillaries and decreases in severely compromised microvessels.

### Capillary permeability in the spinal cord

Efforts to substantiate restoration of BSCB in symptomatic ALS animals through administration of hBMEPCs included imaging of microvascular Evans blue leakage and measurements of EB extravasated into spinal cord parenchyma of 17-weeks-old hBMEPC-treated, media, and control animals.

**Figure 7:**
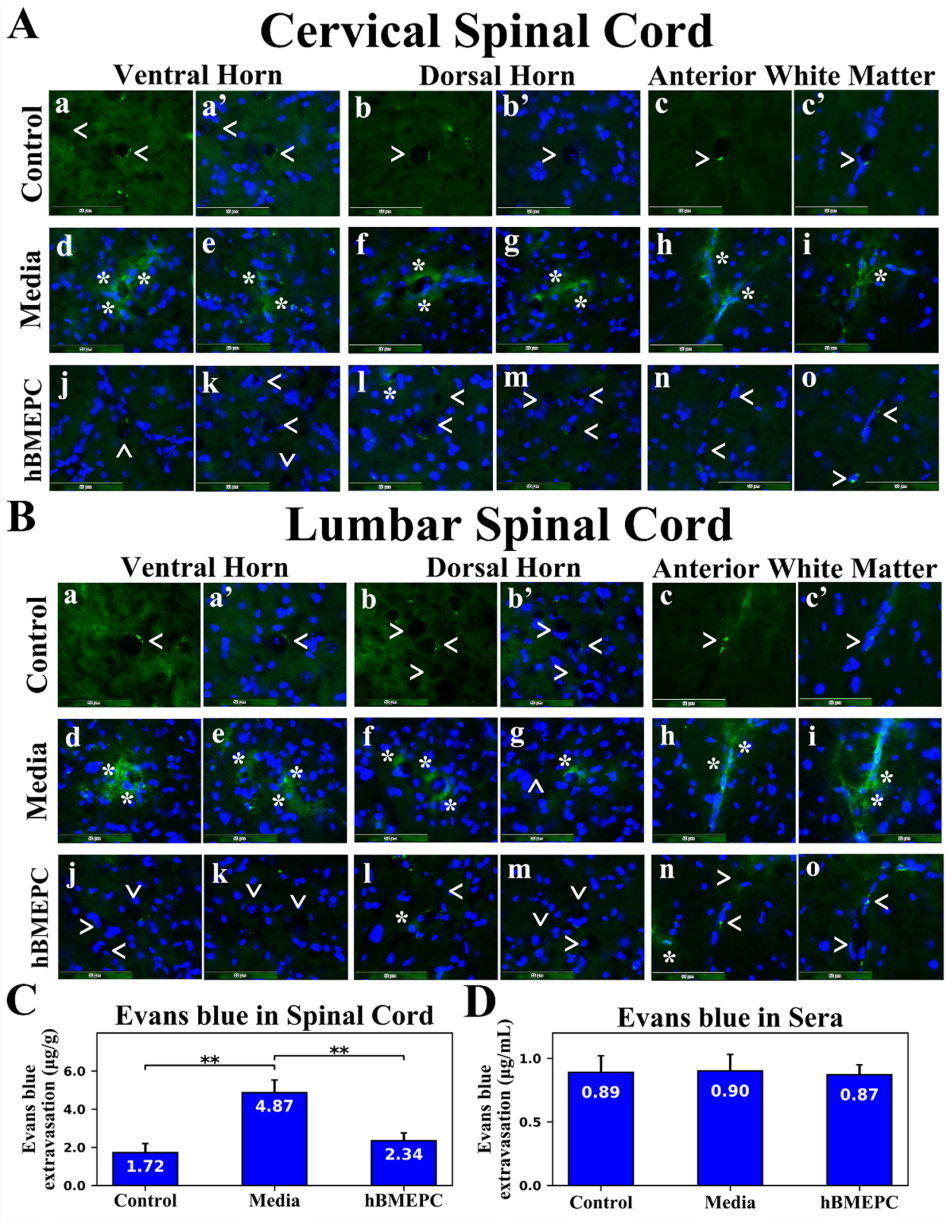
Characteristics of capillary permeability for Evans Blue in the spinal cords of G93A mice. (**A**) In the cervical spinal cord, intravenously injected EB was clearly detected within the blood vessels (green, arrowheads) in the ventral horn (a,a’), dorsal horn (b,b’), and anterior white matter (c,c’) from control mice at 17 weeks of age. Vascular leakage (green, asterisks) of EB, even at some distance from capillaries, was observed in the ventral horn (d,e), dorsal horn (f,g), and anterior white matter (h,i) in cervical spinal cords from media-treated animals of the same age. Cell-treated ALS mice showed substantial reductions of leaky capillaries (green, arrowheads) in the ventral horn (j,k), dorsal horn (l,m), and anterior white matter (n,o). Note, a few capillaries leaking EB (asterisk) were detected in the dorsal horn (**l**) of these animals. The nuclei in a’, b’, c’, d-o are shown with DAPI. Scale bar in a-o is 50 µm. (**B**) Similar to findings in the cervical spinal cord, EB dye was observed intravascularly (green, arrowheads) in the ventral horn (a,a’), dorsal horn (b,b’), and anterior white matter (c,c’) of the lumbar spinal cords from control mice. Significant diffusion of EB (green, asterisks) into the spinal cord parenchyma from numerous blood vessels was detected in the ventral horn (d,e), dorsal horn (f,g), and anterior white matter (**h,i**) from media-treated mice. Extensively extravasated EB was determined at a distance from capillaries in the ventral horn (d) and anterior white matter (**i**). Reduced capillary permeability (green, arrowheads) was shown in the ventral horn (j,k), dorsal horn (l,m), and anterior white matter (**n,o**) from cell-treated mice. Analogous to the cervical spinal cord, some capillaries showed minor EB leakage (asterisks) in the dorsal horn (l) and anterior white matter (n). The nuclei in a’, b’, c’, d-o are shown with DAPI. Scale bar in A-O’ is 50 µm. (**C**) Quantitative analysis of EB extravasation into the spinal cord parenchyma demonstrated significantly higher levels of EB in media-treated mice vs. controls. A significant reduction of extravasated EB into the spinal cords was found in ALS mice after cell transplantation. (**D**) There were no significant differences in EB concentration in sera between control, media, and cell-treated mice. ^**^p < 0.01.

In cervical segments of the spinal cord, Evans blue dye was observed in microvessel lumen of ventral horn (Fig. 7Aa,Aa’), dorsal horn (Fig. 7Ab,Ab’), and anterior white matter (Fig. 7Ac,Ac’) from control mice. Evans blue leaking from vessels was determined in the ventral horn (Fig. 7Ad,Ae), dorsal horn (Fig. 7Af,Ag), and anterior white matter (Fig. 7Ah,Ai) from media animals. Notably, extravasated dye was found at a distance from microvessels in analyzed spinal cord areas of these mice. Substantial reductions of leaky capillaries in the ventral horn (Fig. 7Aj,Ak), dorsal horn (Fig. 7Al,Am), and anterior white matter (Fig. 7An,Ao) were detected in cell-treated ALS mice. Only a few microvessels were observed showing EB leakage in the dorsal horn (Fig. 7Al). Additional immunohistochemical analyses using CD31 to confirm capillary permeability for EB (see Supplementary Fig. 2S) showed distinct capillaries in the ventral (**a, a’, a”**) and dorsal (**b, b’, b”**) spinal horns of controls mice without dye leakage outside microvessels. Media animals demonstrated substantial EB extravasation into tissue parenchyma from CD31 labeled capillaries in the ventral (**c, c’, c”**) and dorsal (**d, d’, d”**) horns. No obvious capillary dye leakage was observed in analyzed cervical spinal cord segments (**e, e’, e”, f, f’, f”**) from cell-treated mice.

In lumbar segments of the spinal cord, Evans blue was observed in microvessels of the ventral horn (Fig. 7Ba,Ba’), dorsal horn (Fig. 7Bb,Bb’), and anterior white matter (Fig. 7Bc,Bc’) of control animals, similar to findings in cervical segments. EB diffused extensively into parenchyma of the spinal cord from many blood vessels into the ventral horn (Fig. 7Bd,Be), dorsal horn (Fig. 7Bf,Bg), and anterior white matter (Fig. 7Bh,Bi) of media mice. Reduced capillary permeability was demonstrated in the ventral horn (Fig. 7Bj,Bk), dorsal horn (Fig. 7Bl,Bm), and anterior white matter (Fig. 7Bn,Bo) from cell-treated mice. Analogous to the cervical spinal cord, some microvessels showed EB leakage in the dorsal horn (Fig. 7Bl) and anterior white matter (Fig. 7Bn). Similarly to the cervical spinal cord, immunohistochemical analyses using CD31 (see Supplementary Fig. 1S) showed distinct capillaries in the ventral (**a, a’, a”**) and dorsal (**b, b’, b”**) spinal horns of controls mice. In some capillaries, dye was determined within capillary lumen (**a, a’, a”**). In contrast, large EB diffusion into spinal cord parenchyma in the ventral (**c, c’, c”**) and dorsal (**d, d’, d”**) horns was detected outside of CD31 identified capillaries in media-treated mice. Cell-treated ALS mice mostly lacked EB leakage in the ventral lumbar spinal cord segment (**e, e’, e”**). Yet, a minor dye leakage was observed in the ventral spinal horn (**f, f’, f”**). Capillary permeability profiles for EB using CD31 immunostaining were analogous in anterior white matter of the cervical and lumbar spinal cords from control, media, and cell-treated mice (data not shown). Measured levels of Evans blue extravasated into parenchyma of the spinal cord revealed significant (p = 0.001) dye content in media (4.87 ± 0.66 µg/g) versus control mouse tissues (1.72 ± 0.48 µg/g) (Fig. 7C). In contrast, dye leakage into spinal cords was significantly (p = 0.004) decreased in hBMEPC-treated ALS animals (2.34 ± 0.42 µg/g) (Fig. 7C). Quantified Evans blue levels in sera served as controls for EB extravasation into the spinal cord tissues and no significant differences in dye concentrations were noted in sera between control (0.89 ± 0.13 µg/mL), media (0.90 ± 0.13 µg/mL), and hBMEPC-treated mice (0.87 ± 0.08 µg/mL), confirming almost identical levels of Evans blue in blood post intravenous administrations (Fig. 7D).

Thus, functional capillary improvement via significant reduction of Evans blue leaking in the spinal cords of G93A animals receiving hBMEPCs corresponds with the ultrastructural restoration of microvasculature in cell-treated mice.

### Effect of hBMEPC transplantation on perivascular astrocytes

**Figure 8:**
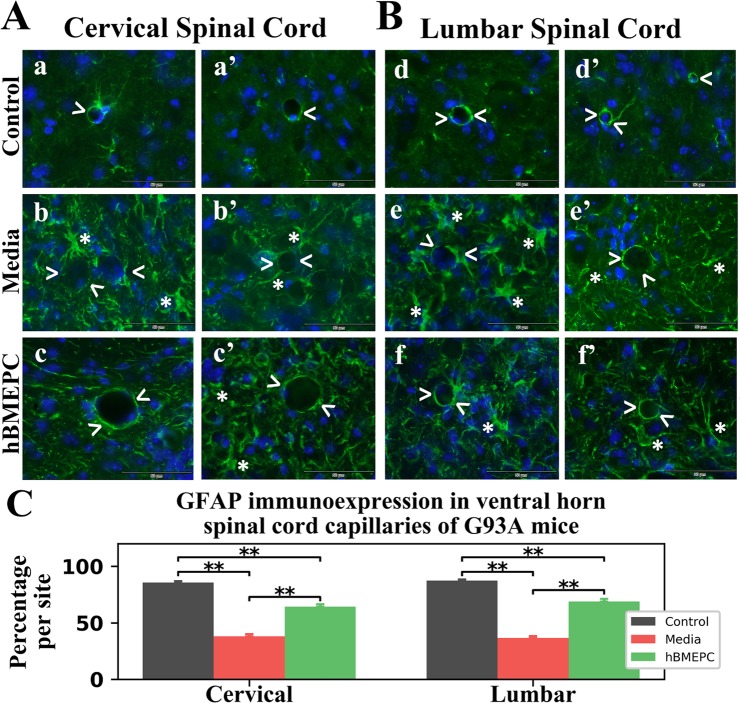
Characteristics of perivascular astrocytes in the cervical and lumbar spinal cord of G93A mice. (**A**) In the cervical spinal cord, immunohistochemical staining of perivascular astrocytes with GFAP antibody (green) in control mice showed normal appearance of delineated astrocytes covering capillaries (a,a’, arrowheads). In media-treated mice, perivascular astrocytes surrounding capillaries were partially revealed (b,b’, arrowheads). Reactive protoplasmic astrocytes (asterisks) near capillaries were also noted in these animals. In cell-treated mice, numerous capillaries demonstrated typical perivascular astrocytes surrounding capillaries (c,c’, arrowheads). Only a few reactive protoplasmic astrocytes (asterisks) near capillaries were observed in these animals. Scale bar in a-c’ is 50 µm. (**B**) In the lumbar spinal cord, perivascular astrocytes fully covered capillaries (d,d’, arrowheads) in control mice, similar to cervical spinal cord findings. (e,e’) Fewer perivascular astrocytes (arrowheads) and notable presence of reactive astrocytes (asterisks) near capillaries were noted in media-injected mice. Many capillaries with typical surrounding perivascular astrocytes (f,f’, arrowheads) were determined in cell-treated mice. Fewer reactive protoplasmic astrocytes (asterisks) were noted. (**B**) Measures of GFAP perivascular immunoexpression showed significantly decreased fluorescence in the cervical and lumbar spinal cords of media-treated mice vs. controls. A significant increase of perivascular GFAP intensity was found in both cervical and lumbar spinal cords from cell-treated vs. media-injected animals. ^**^p < 0.01.

Cervical and lumbar spinal cord perivascular astrocytes from 17-weeks-old hBMEPC-treated, media, and control animals were evaluated by immunohistochemistry, using fluorescent immunostaining of GFAP. GFAP fluorescent images of perivascular astrocytes were analyzed on both sides of the spinal cord ventral horns. Delineated perivascular astrocytes appeared healthy and fully covered capillaries in cervical (Fig. 8Aa,Aa’) and lumbar (Fig. Bd, Bd’) spinal cords of control mice. In media mice, capillaries were partly surrounded by astrocytes as in both cervical (Fig. 8Ab,Ab’) and lumbar (Fig. 8Be,Be’) spinal cords. Additionally, reactive protoplasmic astrocytes proximal to microvessels were observed in these mice. Contrastingly, many microvessels displayed typical perivascular astrocytes in both cervical (Fig. 8Ac,Ac’) and lumbar (Fig. 8Bf,Bj’) spinal cords from hBMEPC-treated animals. A few reactive protoplasmic astrocytes were noted in these mice proximal to capillaries.

Examination of fluorescent GFAP immunoexpression proximal to microvessels revealed analogous significant (p = 0.001) reductions of GFAP intensities in both cervical (38.03 ± 1.88%) and lumbar (36.49 ± 1.76%) spinal ventral horns from media versus control animals: cervical: 85.69 ± 1.22% and lumbar: 87.39 ± 0.95% (Fig. 8C). In the cervical spinal cord, significant (p = 0.001) increases of GFAP perivascular immunoexpression were identified in hBMEPC-treated mice (64.27 ± 2.24%) vs. media animals. Like cervical spinal cord outcomes, ALS mice transplanted with hBMEPCs showed significantly (p = 0.001) elevated GFAP perivascular immunoexpression (68.86 ± 2.39%) in the lumbar spinal cords vs. media-injected mice (Fig. 8C).

Therefore, detailed examination of perivascular GFAP immunoexpression showed re-establishment of perivascular astrocytes in numerous microvessels of both the cervical and lumbar ventral horns in cell-treated mice at 4 weeks post-transplant compared to media-injected mice.

### Effect of hBMEPC transplantation on motor neuron survival

A different collection of randomly selected sections from cervical and lumbar spinal cords of hBMEPC-treated, media, and control animals at 17 weeks of age was assayed with 0.1% cresyl violet to determine condition of motor neurons. Motor neuron status was examined in ventral horns of spinal cord tissues using histology and stereology.

**Figure 9:**
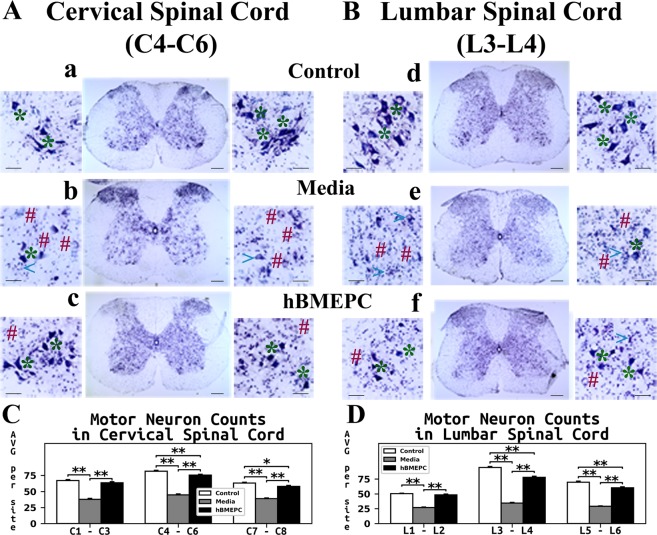
Characteristics of motor neurons in the cervical and lumbar spinal cord of G93A. Histological analysis of motor neurons in (**A**) cervical (C4-C6) and (**B**) lumbar (L3-L4) spinal cords (cresyl violet staining) showed healthy motor neurons in the ventral horns of control animals (a,d, asterisks). Most motor neurons had degenerated or vacuolated in the cervical (b) and lumbar (e) spinal cords of media mice at 17 weeks of age. Cell-treated ALS mice demonstrated substantial motor neuron survival in both cervical (c) and lumbar spinal (f) cord segments at the same age. A few degenerated motor neurons were noted in addition to vacuolated cells. * - healthy motor neuron, # - degenerated motor neuron, <- vacuolated motor neuron. Scale bar in a-f for full coronal spinal cord section is 200 µm and in ventral horn insert is 50 µm. (**C**) Stereological motor neuron counts in discrete levels of the cervical spinal cord showed significantly decreased motor neuron survival in media-injected mice vs. controls. ALS mice receiving cell transplants showed significantly greater motor neuron numbers in all analyzed spinal cord levels compared to media-injected mice. Of note, more surviving motor neurons were found at the enlarged C4-C6 cervical cord level in these animals. (**D**) In the lumbar spinal cord, significantly lower motor neuron numbers at all discrete cord levels were noted in media mice vs. controls, similar to results in the cervical spinal cord. Stereological counts of motor neurons in cell-treated mice showed significantly increased cell numbers in the ventral horns, mainly in the enlarged lumbar level at L3-L4, compared to media-injected animals. ^*^p < 0.05, ^**^p < 0.01.

Typical motor neurons with distinguishable soma and neuritic processes were noted in both cervical (Fig. 9Aa) and lumbar (Fig. 9Bd) spinal cords of control mice. The majority of motor neurons in media animals were degenerated or vacuolated in both analyzed spinal cord segments (Fig. 9Ab,Be). Of note, in media mice, healthy motor neurons were scarce. However, cell-treated mice demonstrated numerous healthy motor neurons with large soma in the cervical (Fig. 9Ac) and lumbar (Fig. 9f) spinal cords. Just a few motor neurons showed signs of degeneration. Also, high densities of segmental-pool motor neurons were noted.

Motor neuron numbers were acquired through stereology at discrete segments of the cervical (C1-C3, C4-C6, and C7-C8) and lumbar (L1-L2, L3-L4, and L5-L6) spinal cord ventral horns. Significantly (p = 0.001) reduced numbers of motor neurons in media mice vs. controls were shown at all analyzed segments (Fig. 9C,D). In cervical segments, cell-treated mice showed significantly (p = 0.001) greater amounts of motor neurons than animals receiving media (Fig. 9C). Higher numbers of motor neurons were noted at the enlarged C4-C6 level in cell-treated mice (75.44 ± 1.33 number/side) vs. media (44.67 ± 1.42 number/side) animals.

Likewise, a significant (p = 0.001) reduction in motor neuron numbers was identified in lumbar spinal cord regions of media versus control mice (Fig. 9D). ALS animals administered with hBMEPCs presented significantly (p = 0.001) increased persistence of motor neurons in all analyzed spinal cord segments vs. media mice, particularly in the enlarged L3-L4 segments. Counts of motor neurons in these lumbar segments from G93A SOD1 mice were: controls – 94.53 ± 1.58, media-treated – 34.41 ± 1.19, and cell-treated – 77.80 ± 1.93 neurons/side (Fig. 9D).

Therefore, histology and stereology data regarding motor neurons stained for Nissl substance in the of the cervical and lumbar spinal cord ventral horns has shown significant survival of motor neurons in hBMEPC-treated mice at 4 weeks after transplantation vs. substantial motor neuron reductions in 17-weeks-old mice receiving media.

## Discussion

This study evaluated the therapeutic approach of human bone marrow-derived endothelial progenitor cells (hBMEPCs) systemically administered into a early symptomatic G93A SOD1 mouse model in towards re-establishing BSCB integrity for enlightenment regarding ALS pathological consequences. Primary findings of the study were that the hBMEPCs effectively: (1) ameliorated disease behavioral outcomes; (2) engrafted into gray and white matter spinal cord capillaries; (3) engrafted into capillaries of brain motor cortex and brainstem; (4) restored capillary ultrastructure; (5) reduced extravasation of Evans blue into parenchyma of the spinal cord; (6) re-established integrity of perivascular astrocyte end-feet; and (7) enhanced motor neuron survival in the spinal cord. These novel findings provide evidence that administration of human bone marrow-derived endothelial progenitor cells benefits repair of the damaged BSCB in ALS, augmenting survival of motor neurons and delaying progression of disease.

Numerous comprehensive reviews^38–42^ have detailed important issues in the endothelial cell transplantation field, including sources of ECs, EC grafting, and the potential of these cells for neurovascular repair, organ vascularization, and/or vehicles for genetic engineering. Supporting the viability of our hBMEPC treatment, systemic transplantation of isolated endothelial progenitor cells (EPCs) from human peripheral blood at same dose of 1 × 10^6^ cells into adult nude mice at 1 hour after transient middle cerebral artery occlusion (tMCAO) showed neurovascular repair, reduced brain cortex atrophy, and improved neurobehavioral outcomes^43^. Importantly, the authors noted that transplanted cells “home” to ischemia-affected areas of the brain within 24 hours of cell administration, promoting brain regeneration. Recently, we reported^44^ that intravenous transplantation of pre-labeled hBMEPCs (4 × 10^6^ cells) into rats at two days post-tMCAO demonstrated robust cell engraftment within microvessels in bilateral striatum and motor cortex and re-established BBB integrity at 5 days after cell administration. Also, many pinocytic vesicles in engrafted cells were shown, indicating high cell functionality. Similar results of human EPC transplantation have been shown in enhancement of tissue neovascularization leading to improved post-ischemia heart and limb functional recovery in animal models^45–47^. Likewise, administration of EPCs derived from cord blood into the ischemic hindlimb in nude rats demonstrated neovascularization and restored blood flow in the injured hindlimb^48^. Thus, EPC administration shows promise as a therapeutic approach for vascular repair. Nevertheless, EPC transplant studies have yet to address BBB/BSCB repair for ALS, or other diseases of neurodegeneration like Alzheimer’s or Parkinson’s with similar barrier pathology^49–53^. In addition, endogenous vascular repair (i.e. endothelial regeneration) typically takes place by replacing dysfunctional ECs with circulated EPCs even in age-related vascular remodeling (reviewed in^54^). Although circulating EPCs can facilitate the endogenous re-endothelialization of damaged vessels, these cells are limited in their ability to replace the damaged endothelium and therefore support from other cell types^55^ may be required for modulation of endogenous vascular homeostasis. We showed^56^ substantially reduced numbers of ECs circulating in ALS-patient peripheral blood at different stages of disease, suggestive of impaired endothelialization. This issue should be taken into consideration for potential endogenous vascular restoration in ALS.

Repairing the BBB/BSCB in amyotrophic lateral sclerosis is essential to protect motor neurons by preventing entry of various detrimental factors, including immune/inflammatory cells, from the systemic compartment to the central nervous system. The existence of BSCB alterations prior to motor neuron degeneration and neuroinflammation in SOD1 rodent mutants modeling ALS^20,21,23,57^ makes barrier restoration even more critical. Since an impaired BBB/BSCB, mainly by alterations of capillary endothelium, is a detrimental element common to both patients and rodent models of disease^15–23,58^, replacement of damaged ECs by administration of “healthy” cells may be a promising new tactic for barrier repair in ALS. Recently, we demonstrated^30–32^ that systemically administering different doses of unmodified hBM34^+^ cells into symptomatic ALS animals had several beneficial effects towards repair of the damaged BSCB leading to delays in disease progression likely caused by increased survival of motor neurons. Although the most positive outcomes such as engraftment of differentiated ECs into spinal cord capillaries^30^ and structural/functional improvement of capillary morphology^31^ were mainly noted in ALS mice receiving the high cell dose, motor enhancement in these mice was primarily determined at 4 weeks post-transplant. Additionally, a large number of capillaries from cervical (17.27 ± 4.75%) and lumbar (9.63 ± 0.99%) spinal cords of high dose hBM34^+^ cell mice still exhibited severe damage. In the present study, the beneficial effect of hBMEPC transplantation at the same dose of 1 × 10^6^ cells on behavioral outcomes in ALS animals was determined as early as 2–3 weeks after cell administration. A smaller percentage of severely damaged capillaries was also detected via electron microscopy in the cervical (9.87 ± 2.72%) and lumbar (5.13 ± 6.29%) spinal cords in mice treated with hBMEPCs vs. hBM34^+^ cell treatment. Importantly, a few mice receiving hBMEPCs were noted showing no ultrastructural evidence of capillaries with severe morphology in the lumbar spinal cords and increased numbers of morphologically normal capillaries to 62.60 ± 4.33% vs. media-treated (23.70 ± 1.91%). Yet, capillary permeability detected by extravasation of EB in the parenchyma of the spinal cord demonstrated significant reductions in G93A animals treated with either cell types vs. media-injected mice at 4 weeks after transplantation. However, the discrepancy in behavioral disease outcomes between mice treated with hBMEPCs and hBM34^+^ cells probably results from engraftment of administered cells onto capillary walls and warrants further discussion.

Results of the present study showed widespread engraftment of transplanted hBMEPCs in numerous gray (anterior and posterior horns) and white (anterior, lateral, and posterior) matter microvessels in both cervical and lumbar spinal cords. Also, engrafted hBMEPCs were determined in regions of the brain associated with degeneration of motor neurons such as the motor cortex (M2/M1), pons, and medulla. These results provide evidence of widespread incorporation of intravenously administered human cells within mouse CNS capillaries leading to structural and functional barrier restoration, at least in the spinal cords. Unfortunately, ultrastructural characteristics of capillary integrity as well as vascular permeability in the brains of hBMEPC-treated vs. media ALS animals were not determined in this study. Nicaise *et al*.^21^ showed significantly increased extravasation of EB, not only in the spinal cord but also in brainstem of a symptomatic G93A rat model of ALS. Further investigations are needed to determine status of the blood-brain barrier prior to and after cell transplantation into ALS mice to confirm the scope of capillary damage and repair in the brain. In addition, analysis of hBM34^+^ cell engraftment was also limited to cervical and lumbar spinal cord ventral horns^30^, making comparisons of post-transplant hBMEPC distribution cumbersome, except in specific areas of the spinal cords. Moreover, hBM34^+^ hematopoietic stem cells require some post-transplant time to differentiate into ECs prior to adhesion and/or engraftment into capillary walls. This issue likely explains the delayed positive effect on motor function of cell-treated mice. In contrast, transplanting restricted-lineage cells, like hBMEPCs, does not require differentiation prior to engraftment, leading to early improvement of behavioral disease outcomes and greater structural capillary restoration. Our other study findings, detection of engrafted hBMEPCs in capillary wall (see Fig. 2A) and substantial reduction of capillary leakage in anterior white matter (see Fig. 7A,B,) are important since the anterior spinal artery supplies blood to the anterior two-thirds of the spinal cord tissue, predominantly to motor areas, including the ventral horns^59^. Thus, “homing” of transplanted hBMEPCs on the luminal capillary surface, as demonstrated in this study, is crucial for re-establishment of the damaged BSCB, and perhaps BBB, maintaining “healthy” blood vessels and CNS homeostasis in ALS.

Proper function of the BBB/BSCB as an essential element of the neurovascular unit depends on reliability of interactions among cellular barrier components, especially, ECs and pericytes. Pericytes proximal to ECs are intimately involved in regulation of capillary permeability and blood flow, as demonstrated in studies using pericyte-deficient mutant mice^60,61^. Importantly, significant reduction of pericyte number and/or pericyte degeneration has been shown in the spinal cords from sporadic ALS patients^16,17,62^. However, pericyte status in animal models of ALS is still unclear. Evidence showing reduced blood flow in the spinal cords of ALS mutant mice even prior to motor neuron degeneration and neuroinflammation^20^ may indirectly suggest early pericyte alteration. In the current study, we analyzed ultrastructural pericyte morphology in the spinal cord of ALS mice at 17 weeks of age. Our results demonstrated pericytes with small cell processes and condensed cytoplasm in the cervical and lumbar spinal cords of media mice, potentially reflecting pericyte degeneration stage. Of note, cell-treated animals showed typical pericyte morphology; only a few pericytes displayed shorter cytoplasmic expansions. Since these data partially characterize pericyte morphology in late symptomatic ALS mice, we are presently performing immunohictochemical pericyte staining in the spinal cord and brain tissues from cell-treated and non-treated G93A animals. Results of this study will be provided in our follow-up manuscript.

Perivascular astrocytes are also imperative cellular component in neurovascular coupling^5,9,14,63,64^. Astrocytic end-feet envelop about 99% of the capillary endothelium^65,66^ and are critical to proper maintenance of ECs, enhancement of endothelial tight junctions, and regulation of neurotransmitter, amino acid, ionic, and water homeostasis in the CNS under physiological condition^6,67–70^. Degenerated perivascular astrocyte end-feet associated with BSCB impairment were determined via electron microscopy in media 17-weeks-old G93A mice in the current study and are supported by our previous findings^18,31^. Also, a more than two fold decrease of capillary delineated astrocytes was noted, as indicated by GFAP immunostaining in both cervical and lumbar spinal ventral horns, similar to our earlier study results^30^. This decline confirmed ultrastructural analysis of perivascular astrocyte status in G93A mice at late stage of disease. Contrastingly, many microvessels covered by representative perivascular astrocytes with high GFAP intensity were identified in spinal cord segments in ALS mice administered with hBMEPCs (present study) or high-cell-dose of hBM34^+^^30^. Of note, perivascular GFAP immunoexpression was greater in hBMEPC-treated mice than in mice administered an equal dose of hBM34^+^ cells. Re-established perivascular astrocyte integrity thus confirmed BSCB repair by hBMEPC treatment. However, the mechanisms of perivascular astrocyte degeneration and restoration remain obscured. Perivascular end-feet of astrocytes proximal to the abluminal capillary membrane support vascular morphology and function. It has been shown that astrocyte-endothelial cell interactions at the molecular level are critical for maintaining barrier integrity in the CNS under physiological and pathological conditions^6,67,71–73^. Controversially, a study by Willis *et al*.^74^ investigating the relationship of focal astrocytes to integrity of the vascular endothelium and maintenance of the BBB concluded that “the BBB can self-repair despite the apparent absence of direct astrocytic-endothelial contact”. We believe that dysfunctional ECs induce perivascular astrocyte degeneration leading to impairment of the BBB/BSCB in ALS. Yet, EC status was not completely determined in the current study. Down-regulation of ZO-1, occludin, and claudin-5 tight junction proteins weakening integrity of the BBB/BSCB and leading to leaky capillaries, has been shown in lumbar spinal cords from mice modeling ALS prior to and during appearance of disease symptoms^20,23^. The expressions of these tight junction proteins after hBMEPC transplantation are currently under investigation and will be reported in a paper currently under development.

Finally, the present study demonstrated significantly increased survival of motor neurons in ALS animals that received hBMEPC transplants through examinations of histology and stereology data from individual spinal cord segments of ventral horns at 17 weeks of age. Higher motor neuron numbers were determined from enlarged C4-C6 cervical and L3-L4 lumbar spinal cord segments versus animals receiving media, similar to results obtained in post-treatment mice administered the large dose of hBM34^+^^30^ cells. However, more motor neurons were found at the C4-C6 spinal cord segments in mice treated with hBMEPCs (75.44 ± 1.33) than the same dose of hBM34^+^ cells (58.94 ± 1.67) and near equivalent numbers were noted at L3-L4 level (hBMEPCs: 77.80 ± 1.93; hBM34^+^ cells: 74.84 ± 2.10). Taking into account that impaired motor function in the G93A SOD1 mouse becomes initially apparent in the hindlimbs (innervated from lumbar region of spinal cord) and following motor deficiencies are detected in the front limbs (innervated from cervical area of spinal cord)^75^, our data from the current and previous^30^ studies support this differential limb involvement with larger numbers of motor neurons in the cervical ventral horn (~45/side) vs. lumbar (~34/side) enlarged segments in media mice at 17 weeks of age. Determining survival of motor neurons at different segmental spinal cord levels is important to evaluate cell transplant effects and prove superior motor neuron survival in both cervical and lumbar spinal cords of hBMEPC-treated mice.

Lastly, neuroinflammation has been implicated in motor neuron degeneration and is considered a significant component in ALS pathogenesis^76–82^. It has been shown that numerous peripheral factors, including immune/inflammatory cells, readily enter the CNS parenchyma and may exacerbate motor neuron dysfunction^77,81,83–90^. Repair of the BBB/BSCB could block infiltration of detrimental peripheral effectors preventing motor neuron damage and serve as one mechanism for neuroprotection in ALS. Supporting this suggestion, we showed enhanced survival of cervical and lumbar spinal cord motor neurons in hBMEPC-treated ALS mice. However, additional confirmation of reduced neuroinflammation is needed through evaluation of post-treatment parenchymal astrocytes and microglia status. Our study is in progress.

Furthermore, alternative therapeutic approaches leading to the BBB/BSCB restoration in ALS might be considered. These interventions were previously discussed in detail^30^. Particularly, activated protein C (APC) has multiple actions providing cytoprotective and anti-inflammatory effects^91–94^. It has been shown that administration of APC analogs into symptomatic G93A mice delayed microglia activation and reduced capillary leakage leading to slower disease progression and extended mouse lifespan^57,95^. Since barrier repair in ALS was achieved by anti-inflammatory drug action, combined therapy with APC and hBMEPCs might be attractive.

In conclusion, intravenous administration of bone marrow-derived endothelial progenitor cells (hBMEPCs) into symptomatic ALS mice leads to improved motor function and prolonged motor neuron survival. These benefits may have been promoted by widespread engraftment of transplanted cells into microvessel walls in gray and white matter spinal cords leading to BSCB repair. Ultrastructural capillary restoration, significant decrease of capillary leakage, and enhancement of perivascular end-feet eminence strongly support our suggestion. Also, detection of engrafted hBMEPCs in the brain areas of motor neuron degeneration such as motor cortex and brainstem supports the efficacy of intravenous delivery for pervasive cell distribution. Although various studies are needed towards evaluating mechanisms responsible for transplanted cell actions, such as cell adherence to the capillary wall or potential secretion of specific factors for maintenance endothelium, our study results indicate that transplanting human hematopoietic cells derived from a restricted lineage, like endothelial progenitor cells, improves restoration of the BSCB in ALS mice versus non-differentiated stem cells. However, since severe damage was identified in some microvessels via electron microscopy in cell-treated animals, a series of smaller cell doses administered into symptomatic ALS mice may effectively aid EC reparative processes during progression of this disorder. Although the presence of transplanted cells in blood circulation of mice shown in this study may support continuing processes re-establishing BSCB integrity, the lack of capillary adherence and/or engraftment of these cells should be investigated. We would also like to emphasize, that from a translational viewpoint, initiation of cell treatment at the symptomatic disease stage offered robust restoration of BSCB integrity and shows promise as a future clinical therapy for ALS.

## Supplementary information

Supplemental figures

## Data Availability

The datasets generated during and/or analyzed during the current study are available from the corresponding author on reasonable request.
