# Supplementary material for: Human Bone Marrow Endothelial Progenitor Cell Transplantation into Symptomatic ALS Mice Delays Disease Progression and Increases Motor Neuron Survival by Repairing Blood-Spinal Cord Barrier
Source: Sci Rep. 2019 Mar 27;9:5280. doi: 10.1038/s41598-019-41747-4 (PMC6437219; doi:10.1038/s41598-019-41747-4)
Supplement: Supplementary file 1 — Supplemental figures [file 41598_2019_41747_MOESM1_ESM.pdf]

Human Bone Marrow Endothelial Progenitor Cell Transplantation into Symptomatic ALS Mice Delays Disease Progression and Increases Motor Neuron Survival by Repairing Blood-Spinal Cord Barrier

Svitlana Garbuzova-Davis, Crupa Kurien, Edward Haller, David J. Eve, Stephanie Navarro, George Steiner, Ajay Mahendrasah, Surafuale Hailu, Mohammed Khatib, Kayla J. Boccio, Cesario V. Borlongan, Harry R. Van Loveren, Stanley H. Appel, Paul R. Sanberg

Characteristics of human bone marrow derived endothelial progenitor cells *in vitro*

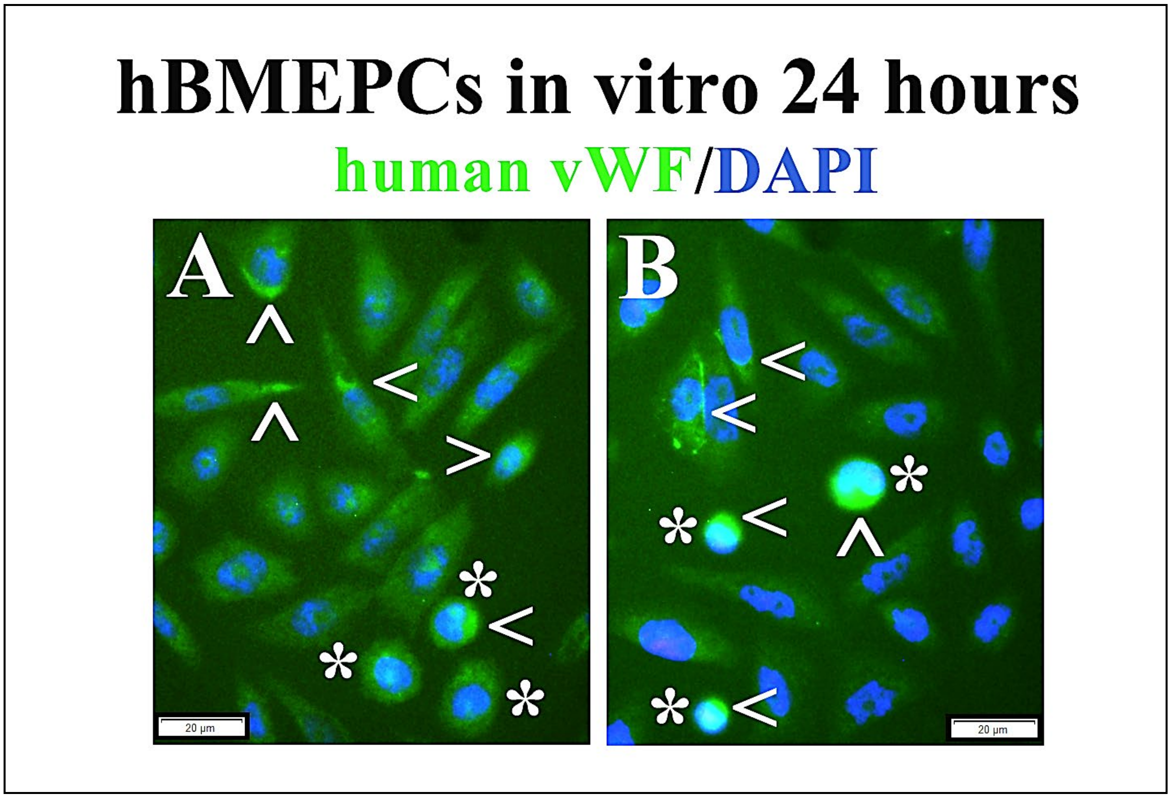

Supplementary Figure 1S. In culture of hBMEPCs for 24 hours, immunocytochemical staining using human specific vWF antibody showed antigen immunoexpression (green, arrowheads) in all cells at different degrees and localizations (**A** and **B**). There are two distinct cell morphologies: rounded (asterisks) and elongated. The nuclei in all images are shown with DAPI (blue). Scale bar in A and B is 20 µm.

Immunohistochemical characteristics of capillary permeability for Evans Blue in the cervical spinal cords of G93A mice

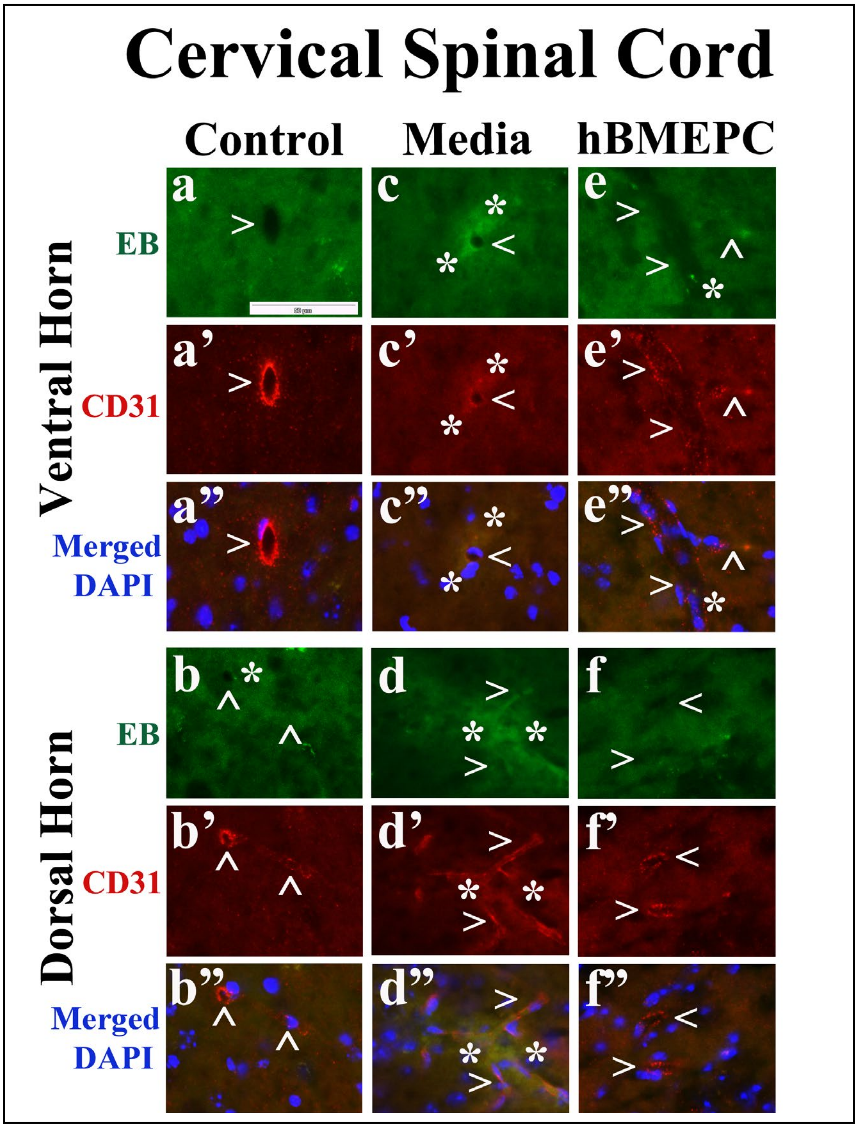

Supplementary Figure 2S. Immunohistochemical staining for CD31, an endothelial cell marker, was performed to visualize capillary permeability for Evans Blue (EB) in the cervical spinal cords of G93A mice at 17 weeks of age. In control mice, capillaries were well identified by CD31 (red, arrowheads) in the ventral (**a, a', a"**) and dorsal (**b, b', b"**) horns. EB leakage outside of microvessels was not determined; only small dots of dye (**b**, green, asterisk) were observed within capillary lumen. Substantial EB extravasation (green, asterisks) into tissue parenchyma was detected in the ventral (**c, c', c"**) and dorsal (**d, d', d"**) spinal horns in media mice. Cell-treated mice showed no obvious capillary dye leakage (arrowheads) in analyzed spinal cord segments (**e, e', e", f, f', f"**). A few vessels displayed luminal EB dye (**e, e"**, asterisks). The nuclei in a", c", e", b", d", f" are shown with DAPI (blue). Scale bar (50 μm) shown in a is same for all images.

Immunohistochemical characteristics of capillary permeability for Evans Blue in the lumbar spinal cords of G93A mice

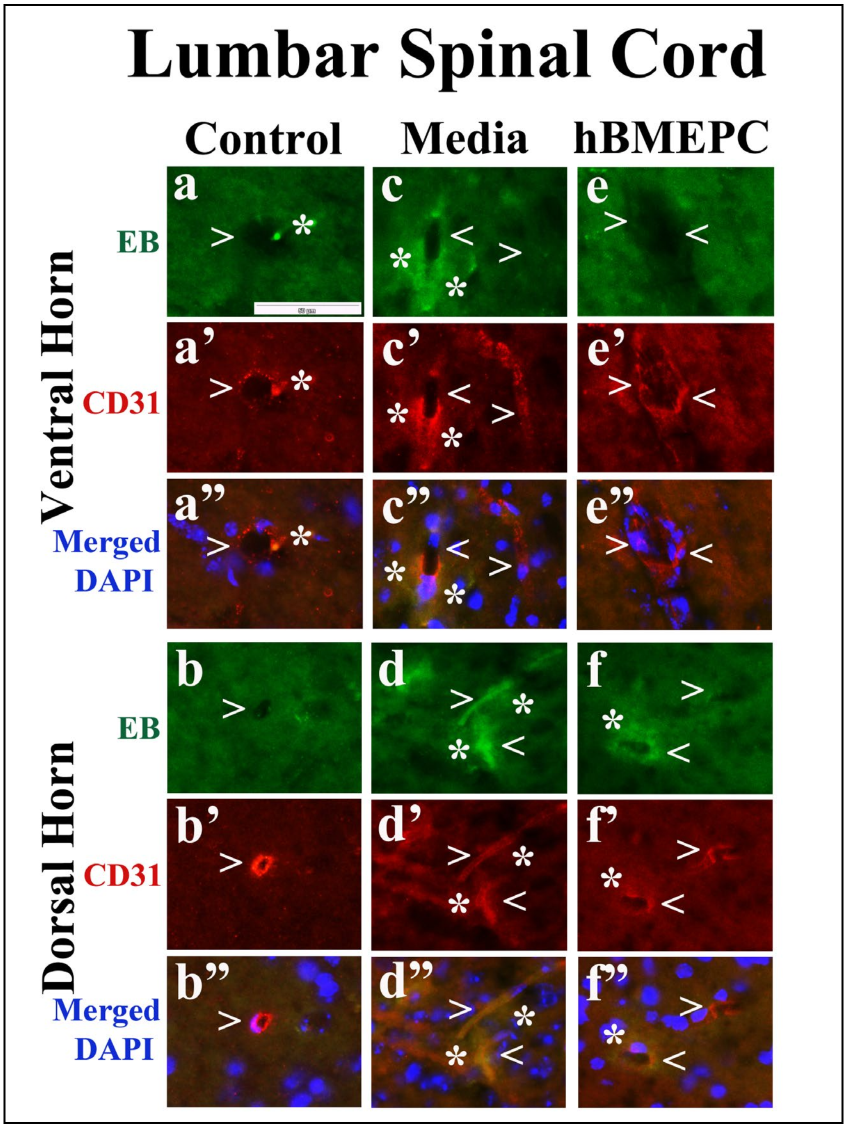

Supplementary Figure 3S. Immunohistochemical staining for CD31, an endothelial cell marker, was performed to visualize capillary permeability for Evans Blue (EB) in the lumbar spinal cords of G93A mice at 17 weeks of age. Control mice showed distinct capillaries (red, arrowheads) in the ventral (**a, a', a"**) and dorsal (**b, b', b"**) horns. EB was determined within capillary lumen (**a, a', a"**, green, asterisk). Large EB diffusion (green, asterisks) into tissue parenchyma in media mice was detected in the ventral (**c, c', c"**) and dorsal (**d, d', d"**) spinal horns similar to findings in the cervical spinal cord. ALS mice with cell treatment showed mainly lack of EB leakage in the ventral horn (**e, e', e"**, arrowheads). Some vessels demonstrated minor dye leakage in the dorsal spinal horn (**f, f', f"**, asterisks). The nuclei in a", c", e", b", d", f" are shown with DAPI (blue). Scale bar (50 μm) shown in a is same for all images.
